# Genome-wide Analysis of Simultaneous GATA1/2, RUNX1, FLI1, and SCL Binding in Megakaryocytes Identifies Hematopoietic Regulators

**DOI:** 10.1016/j.devcel.2011.04.008

**Published:** 2011-05-17

**Authors:** Marloes R. Tijssen, Ana Cvejic, Anagha Joshi, Rebecca L. Hannah, Rita Ferreira, Ariel Forrai, Dana C. Bellissimo, S. Helen Oram, Peter A. Smethurst, Nicola K. Wilson, Xiaonan Wang, Katrin Ottersbach, Derek L. Stemple, Anthony R. Green, Willem H. Ouwehand, Berthold Göttgens

**Affiliations:** 1 Department of Haematology, Cambridge Institute for Medical Research, University of Cambridge, Cambridge CB2 0XY, UK; 2 Department of Haematology, NHS Blood and Transplant Centre, University of Cambridge, Cambridge CB2 0PT, UK; 3 Wellcome Trust Sanger Institute, Hinxton, Cambridge CB10 1SA, UK

## Abstract

Hematopoietic differentiation critically depends on combinations of transcriptional regulators controlling the development of individual lineages. Here, we report the genome-wide binding sites for the five key hematopoietic transcription factors—GATA1, GATA2, RUNX1, FLI1, and TAL1/SCL—in primary human megakaryocytes. Statistical analysis of the 17,263 regions bound by at least one factor demonstrated that simultaneous binding by all five factors was the most enriched pattern and often occurred near known hematopoietic regulators. Eight genes not previously appreciated to function in hematopoiesis that were bound by all five factors were shown to be essential for thrombocyte and/or erythroid development in zebrafish. Moreover, one of these genes encoding the PDZK1IP1 protein shared transcriptional enhancer elements with the blood stem cell regulator TAL1/SCL. Multifactor ChIP-Seq analysis in primary human cells coupled with a high-throughput in vivo perturbation screen therefore offers a powerful strategy to identify essential regulators of complex mammalian differentiation processes.

## Highlights

► Genome-wide TF binding analysis in primary human megakaryocyte ► Regulatory network reconstruction from TF, epigenetic, and expression data ► Simultaneous binding of five TFs marks hematopoietic regulators ► Five-factor binding reveals eight genes essential for zebrafish blood development

## Introduction

Complex gene-regulatory networks control all metazoan development (Davidson, 2001). Transcription factors (TFs) and the *cis*-regulatory sequences to which they bind form the building blocks of these gene-regulatory networks. Connectivity in these networks has historically been determined using individual gene-based assays or inferred from expression profiling. However, gene by gene characterization of regulatory elements is slow and inference of regulatory relationships based on variations in gene expression alone cannot readily differentiate between direct and indirect interactions. New genome-wide technologies permit potentially rapid access to all regions bound by a given factor. However, binding of a factor does not necessarily equate with direct regulation of nearby genes. New strategies are therefore required to identify the subsets of binding events that are central to regulatory network function.

Hematopoiesis has long served as a model process for studying stem cells (Orkin and Zon, 2008). One of the earliest branch points during hematopoietic differentiation is the specification of the megakaryocyte (MK)/erythroid progenitor (MEP) (Adolfsson et al., 2005). Subsequent formation of immature MKs leads to the production of large numbers of anucleate platelets. As the second most abundant cell in the blood, their primary role is to maintain hemostasis and instigate wound healing on vascular damage.

Being derived from a common precursor, the MK and erythrocyte lineages share a number of TFs critical for their development, including GATA1, FOG1, SCL, and GFI1b. Other factors, like EKLF (erythroid) and GABPα, FLI1, and RUNX1 (megakaryocytic) have been implicated in the bifurcation of these lineages. Analysis of cell-type specific regulatory elements is consistent with the notion that combinations of these TFs constitute lineage-specific regulatory codes. For example, sites for GATA1 and SCL are found in several erythroid specific enhancers (Anderson et al., 1998; Wadman et al., 1997) and several enhancers active in MKs contain GATA and ETS binding sites (Minami et al., 1998; Yamaguchi et al., 1998).

We have recently reported genome-wide combinatorial interactions for ten key regulators of hematopoietic stem/progenitor cells in the multipotent mouse hematopoietic progenitor cell line 7 (HPC-7) (Wilson et al., 2010a), which revealed combinatorial interactions between a heptad of TFs (Scl, Lyl1, Lmo2, Gata2, Runx1, Erg, Fli1). Subsequent in vivo validation confirmed a previously unrecognized synergism between the three central regulators of blood stem cell development Scl, Gata2, and Runx1. Another elegant study examined the dynamic binding and long-distance regulation by multi-TF complexes during erythroid maturation using a mouse erythroleukemia cell line model (Soler et al., 2010). Furthermore, recent studies have extended genome-wide analysis of key hematopoietic transcription factors to human leukemia cell lines (Fujiwara et al., 2009; Pencovich et al., 2011). However, all the above studies were performed in transformed cell lines. Any findings therefore come with the many caveats associated with the unphysiological nature of immortalized cells. Here we report the genome-wide binding of five key hematopoietic regulators in primary human MKs. Knockdown in zebrafish of eight genes, regulated by all five TFs and with no known function in hematopoiesis, showed all to be essential for thrombocyte and/or erythroid development, suggesting that multifactor chromatin immunoprecipitation sequencing (ChIP-Seq) combined with high-throughput zebrafish screening is a widely applicable strategy to identify regulators of complex mammalian developmental processes.

## Results

### Genome-Wide Analysis of GATA1, GATA2, RUNX1, FLI1, and SCL Binding in Primary Human Megakaryocytes

**Figure 1:**
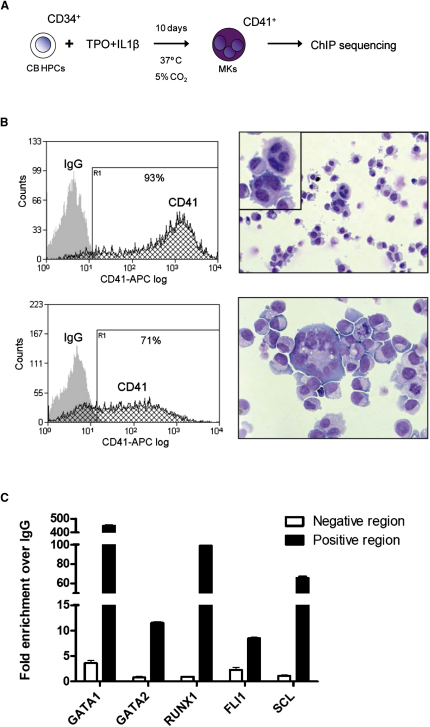
GATA2, RUNX1, FLI1, and SCL Bind to the *RUNX1* +23 and GATA1 to the *SCL* +40 Enhancer in Primary Megakaryocyte Cultures (A) Cord blood CD34^+^ hematopoietic progenitor cells were differentiated into CD41^+^ MKs using a 10 day culture in serum-free medium containing TPO and IL-1β. Subsequently ChIP for GATA1, GATA2, RUNX1, FLI1, and SCL was performed. (B) After culture, 71%–93% of cells were CD41^+^ as determined by flow cytometry (IgG is depicted in gray and CD41 stain in black). A cytospin and modified Wright's stain revealed the presence of pro-MKs with nuclear separation in between megakaryoblasts. (C) ChIP material was validated by real-time PCR using primer pairs for regions known to be bound (positive region) or not bound (negative region). For GATA2, RUNX1, FLI1, and SCL, the *RUNX1* intron 1 +23 enhancer was used as a positive region with the *RUNX1* +31kb as corresponding negative region. For GATA1, the *SCL* +40 enhancer was used as a positive region with the *SCL* −16 as negative region (mean + SD; see Supplemental Experimental Procedures for details).

To determine genome-wide TF binding, primary MKs were cultured from cord blood CD34^+^ hematopoietic progenitor cells (HPCs; ≥98% pure) for 10 days in the presence of thrombopoietin and interleukin-1β (Figure 1A). 3.3 million CD34^+^ cells produced a total of 94.7 million primary cells, sufficient for comprehensive genome-wide analysis. After culture, 71%–93% of cells expressed CD41 (Figure 1B) and 20%–56% expressed the more mature marker CD42a (data not shown). Cytospin and modified Wright's stain demonstrated that most cells were at the megakaryoblast stage, however, some pro-MKs with nuclear separation were also seen (Figure 1B). Greater than 90% of cells tested negative for CD34 expression and few cells expressed markers of other lineages (CD11c, 0.4%–4.1%; CD14, 2.4%–2.7%; CD15, 0.7%–2.3%; CD66a, 0.5%–1%; data not shown).

ChIP-Seq technology was then used to generate genome-scale catalogs of sequences bound by GATA1, GATA2, RUNX1, FLI1, and SCL in primary human MKs. Before sequencing, ChIP material was validated by quantitative polymerase chain reaction (PCR). We have previously shown that the *RUNX1* +23 enhancer is bound by GATA2, RUNX1, FLI1, and SCL in mouse progenitor cells (Wilson et al., 2010a), and the *SCL* +40 enhancer by GATA1 in erythroid cells (Ogilvy et al., 2007). As shown in Figure 1C, significant binding of the relevant factors to these two regions was also observed in primary human MKs.

**Figure 2:**
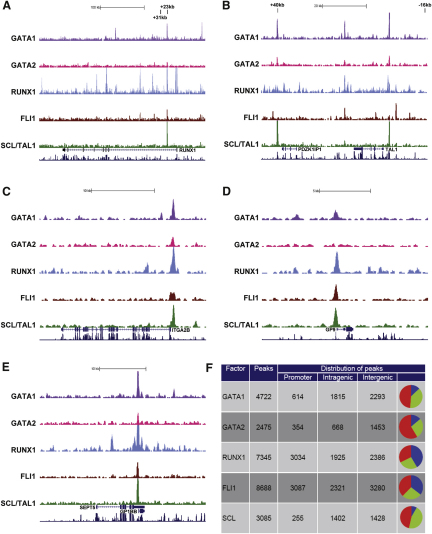
GATA1, GATA2, RUNX1, FLI1, and SCL Binding to the Human Megakaryocyte Genome (A–E) Raw ChIP-Seq read data was transformed into density plots and displayed in the UCSC genome browser above the tracks for gene structure and vertebrate homology. The vertical viewing range was set at 0–50. Black vertical bars show the location of the PCR primers used to validate the ChIP material. Shown here are the loci *RUNX1* (A), *SCL/TAL1* (B), *ITGA2B* (CD41) (C), *GP9* (CD42a) (D), and *GP1BB* (CD42c) (E). (F) Each peak was allocated to be either within a promoter, intragenic, or intergenic region. The pie chart shows the distribution of the peaks across those three categories (blue, green, and red, respectively). See also Figure S1.

The ChIP material was sequenced yielding between 10.3 million and 14.1 million mappable reads for each factor. Visualization of the raw data across the *RUNX1* and *TAL1* loci reproduced the PCR results with specific enrichment of the *RUNX1* +23 and *SCL* +40 regions (Figures 2A and 2B, respectively). Inspection of several gene loci for MK-affiliated genes showed that (1) the *ITGA2B* locus (coding for integrin αIIb, the CD41 marker used to determine the purity of the MK cultures) is bound by all five TFs (Figure 2C); (2) *GP9* coding for the MK-specific protein GPIX is bound by all but GATA2 (Figure 2D); and (3) *GP1BB* is bound by all five TFs just like CD41 (Figure 2E).

To comprehensively identify all binding peaks across the genome, we generated a control IgG ChIP sample to permit the use of software tools specifically designed to eliminate artifactual peaks found in both test and control samples, which allowed us to identify 4722 peaks for GATA1, 2475 peaks for GATA2, 7345 peaks for RUNX1, 8688 peaks for FLI1, and 3085 peaks for SCL (Figure 2F). Over 33% of RUNX1 and FLI1 peaks were situated in promoter regions, whereas for GATA1, GATA2, and SCL this did not exceed 14%.

### Analysis of Combinatorial Interactions Highlights a Preference for All Five Factors Binding Together

**Figure 3:**
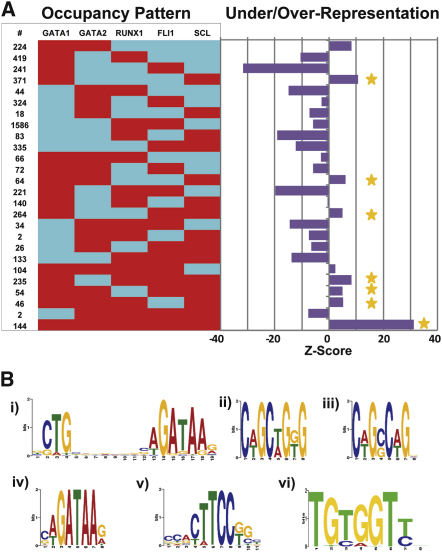
Analysis of Combinatorial Binding Identifies Prevalent Patterns and Suggests Indirect Recruitment of SCL and RUNX1 (A) The number of peaks for all 26 combinations involving binding of two or more factors are shown on the left of the figure (red = bound, blue = not bound). Z scores on the right indicate significance of deviation between observed and expected instances for all 26 binding patterns. Seven of eight combinations containing GATA1 and SCL were overrepresented (marked with a yellow star). (B) De novo motif discovery on sequences bound only by GATA1 and SCL recovers a typical SCL/GATA1 composite binding motif (i), the SCL binding E-box motif (ii), and a motif resembling an E-box (iii). All regions bound by GATA1 and SCL plus one or more of the other factors, recovered GATA, ETS, and RUNX consensus sequences (iv, v, and vi, respectively). See also Tables S1 and S2 and Figure S2.

To obtain new insights into combinatorial TF interactions, we next asked whether the frequency of colocalization of specific combinations of TFs on the same target regions might indicate preferential coregulatory activities. To this end, we determined the number of overlapping peaks for all 26 possible combinations involving binding of two or more factors (Figure 3A, left; see Tables S1 and S2 available online for all peak coordinates and candidate target genes). To address statistical significance, we used a lower end estimate of 80,000 regions potentially available for binding (Boyle et al., 2008; McDaniell et al., 2010), determined the expected frequencies for all 26 binding patterns and calculated significance of deviation between observed and expected values (Figure 3A, right).The most underrepresented pattern was binding by GATA1 and FLI1 without any other factor, suggesting that binding by GATA1 and FLI1 in the absence of larger multi-TF-complexes is not favored. Interestingly, seven of eight combinations involving GATA1 and SCL were overrepresented pointing to the importance of this pairing in megakaryopoiesis (Figure 3A). By far the most significant combinatorial pattern was binding of all five factors together suggesting an important role for this complex in MK transcriptional control. Of note, there was only a minor overlap between the peaks identified here when compared to those from a recently published study where four of the five factors were analyzed in a mouse hematopoietic progenitor cell line (Figure S1), suggesting that cell-type specific binding previously reported for individual TFs (Heinz et al., 2010) extends to multifactor combinatorial binding.

We next used sets of regions with particular TF occupancy patterns for de novo motif discovery, which recovered the expected consensus binding motifs (Figure 3; see Figure S2 for expected consensus motif sequences). For example, analysis of sequences bound only by SCL and GATA1 recovered a typical SCL/GATA1 composite binding motif (Figure 3Bi), also seen in recent ChIP-Seq studies of Scl in erythroid cells (e.g., Kassouf et al., 2010 and Soler et al., 2010) and now recognized as the likely docking site for Scl/Gata complexes. This analysis also recovered the SCL binding E-box motif (Figure 3Bii) as well as a motif resembling an E-box, but not present in current collections of transcription factor consensus binding sites (Figure 3Biii). Analysis of other subsets such as all regions bound by SCL and GATA1 plus one or more of the other three factors recovered additional expected motifs such as GATA, ETS, and RUNX consensus sequences (e.g., Figures 3Biv–3Bvi), thus demonstrating that expected motifs were present in the regions identified by ChIP-Seq analysis.

### Only a Subset of Peak Occupancy Patterns Correlates with Megakaryocyte-Specific Gene Expression

**Figure 4:**
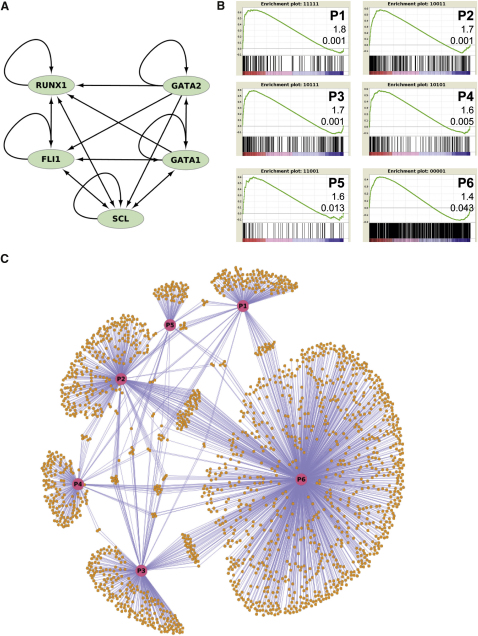
A Regulatory Network Model for Megakaryocyte-Specific Expression (A) GATA1, GATA2, RUNX1, FLI1, and SCL form a densely connected core circuit replete with positive feedback loops. (B) Gene set enrichment analysis (GSEA) shows highly significant enrichment for expression in MKs in 6 of the 31 possible combinatorial binding patterns (see Table S3 for full results). Shown are plots of the running sum for the genes regulated by these six patterns in relation to all genes ranked for expression in MKs compared to six other human blood cell types (Watkins et al., 2009) (CD4, CD8, CD14, CD19, CD56, CD66b, and erythroblasts). The six occupancy patterns significant in GSEA are labeled P1 to P6 for ease of representation in (C). The normalized enrichment score and false discovery rate q value are also shown. (C) A regulatory network model for gene expression in MKs regulated by the six occupancy patterns showing significant correlation by GSEA. Shown are the links to downstream target genes based on peak-to-gene mapping for the patterns P1–P6 (B). Although some genes are regulated by multiple patterns, most downstream effector genes are controlled by a single node. See also Table S3 and Figure S3.

Having established the potential importance of combinatorial interactions between GATA1, GATA2, RUNX1, FLI1, and SCL, we next investigated the connectivity within the core network formed by these five factors. As shown in Figure 4A, all five TFs are characterized by a positive autoregulatory feedback loop, and in addition contain extensive cross-regulatory links, thus resulting in the formation of a densely connected core circuit. Studies in lower model organisms have suggested that regulatory networks commonly consists of densely connected TF core circuits that control large numbers of tissue-specific effector proteins (enzymes, structural proteins, etc.) (Davidson, 2010; Roy et al., 2010). With occupancy patterns being nonrandomly distributed (Figure 3A), we next investigated the nature of the candidate target genes for all possible 31 occupancy patterns, and therefore mapped TF binding peaks to nearby genes thus generating 31 target gene lists (Table S2). To identify those sets of target genes most likely to be important for the megakaryocyte phenotype, we next performed gene set enrichment analysis (GSEA), which demonstrated that six occupancy patterns were associated with genes highly enriched for MK-specific expression (Figure 4B and Table S3). Of note, four of these six peak occupancy patterns (P1, P2, P3, P5) had also been found to occur much more frequently than expected by chance (see Figure 3A). This convergence between statistical analysis of peak pattern frequency and GSEA analysis of neighboring genes therefore suggests that key aspects of the megakaryocyte transcriptional program can be captured from comprehensive analysis of our data set.

Of note, none of the 31 gene lists showed significant correlation with genes specifically repressed in MKs, thus suggesting that the five-factor core circuit analyzed here largely functions as a positive regulator of MK expression. To further explore this notion, we performed additional ChIP-Seq experiments to generate genome-wide maps indicative of active and repressed chromatin. Trimethylation of histone H3 lysine 4 (H3K4me3) was used to map active promoters, histone H3 acetylation (H3acet) for active promoters as well as more distal elements, and trimethylation of histone H3 lysine 27 (H3K27me3) as a mark for repressed chromatin. All three data sets displayed the expected patterns of enrichment (Figure S3A). We therefore proceeded to categorize all human promoters into three categories (inactive, bivalent and active) based on their pattern of histone modification (Figure S3B). As expected, when gene expression in the three categories was examined, the highest level of expression was found in the category of “active” promoters (Figure S3C). Moreover, the fraction of promoters bound by the five transcription factors was lowest for the inactive promoters, at an intermediate level for bivalent promoters and highest for the active promoters (Figure S3D). Genome-wide analysis of chromatin status was therefore consistent with the notion that the core TF circuit defined here is predominantly involved in positive regulation of gene expression.

With analysis of combinatorial binding patterns, gene set enrichment and chromatin analysis all converging on a consistent role for the GATA1/GATA2/RUNX1/FLI1/SCL core circuit within wider MK transcriptional programs, we next developed a regulatory network model (Lin et al., 2010) illustrating the connectivity of all genes controlled by TF peaks correlating with MK-specific expression (e.g., the gene sets corresponding to patterns P1 to P6 from Figure 4B). As illustrated in Figure 4C, target genes controlled by the six different types of peaks are largely nonoverlapping. This analysis therefore suggests that control of MK effector genes mainly occurs via a single regulatory element whereby several distinct combinations of GATA1/GATA2/RUNX1/FLI1/SCL can be involved in contributing to MK-specific expression. Taken together, comprehensive bioinformatic analysis has allowed us to generate a regulatory network model for megakaryocyte-specific expression with a densely connected core circuit controlling several hundred effector genes through a relatively shallow hierarchy, a network architecture recently predicted to be common for tissue-specific gene expression programs (Davidson, 2010). It is important to note, however, that not unexpectedly, the current five-factor network model does not capture MK-specific expression in its entirety. Of the top 200 megakaryocyte-specific genes, 67 form part of the current network model, thus suggesting that alternative TF combinations mediating MK-specific expression remain to be discovered.

### In Vivo Validation of Candidate Target Genes in Zebrafish Identifies Eight Hematopoietic Regulators

With all five factors binding together being the most significant combinatorial pattern in our data set (Figure 3A) and the most highly enriched for MK-specific genes (Figure 4B and Table S3), we hypothesized that concurrent binding by these five factors represented an important control mechanism for hematopoietic development. To explore this hypothesis further, we used the genomic regions enrichment of annotations tool (GREAT) (McLean et al., 2010), which defines genomic neighborhoods for TF-bound peaks by attaching weights to flanking genes based on their distance to the peak. Weighted gene lists are then used to identify overrepresented biological functions. GREAT analysis for the 144 regions bound by all five TFs showed strong enrichment for a number of phenotypes, all of which were hematopoietic and included decreased platelet count (Table 1). Taken together therefore, concurrent binding of GATA1, GATA2, RUNX1, FLI1, and SCL highlights a set of genes highly enriched for known (and potentially unidentified) regulators of hematopoiesis in general and of megakaryopoiesis more specifically.

**Figure 5:**
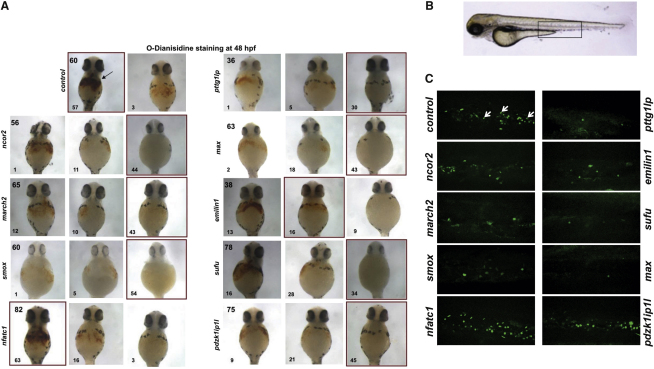
In Vivo Morpholino Screen in Zebrafish Identifies Eight Regulators of Hematopoiesis (A) MOs were injected into one-cell stage embryos and number of erythrocytes assessed with o-dianisidine staining. For eight genes, injection of MOs resulted in a reduced number of blood cells in circulation at 48 hr postfertilization (hpf). For clarity representative images illustrating three different phenotypes (unaffected [left], mild [middle], and severe [right]) are shown with the number of embryos in each group indicated in the lower, left corner. The number in the upper left corner of the very left image shows the total number of embryos used in each experiment. The predominant phenotype is framed in red. Black arrow indicates hemoglobin staining in the control. (B) Wild-type zebrafish embryo at 72 hpf. Confocal images presented in (C) were taken of the caudal hematopoietic tissue, indicated by the boxed region. (C) MOs were injected into the one-cell stage transgenic *Tg*(*cd41*:*EGFP*) zebrafish embryos and assayed for their effect on the number of presumed hematopoietic stem cells (*cd41^low^*) and thrombocytes (*cd41^high^*; white arrows) at 72 hpf. For *march2* (n = 44), *max* (n = 63), *smox* (n = 60), *pttg1lp* (n = 50), *emilin1* (n = 65), and *sufu* (n = 65), a severe decrease of *cd41* positive cells was observed. *Ncor2* (n = 53) depletion resulted in a mild phenotype, and *pdzk1ip1l* (n = 49) and *nfatc1* (n = 60) MO injected embryos showed no phenotype. See also Tables S4–S7 and Figure S4.

To further explore the biological significance of the subset of genes bound by all five TFs, we performed functional studies using zebrafish as a model for high-throughput in vivo validation. Mapping peaks to genes identified 151 candidate target genes for the 144 regions bound by all five TFs of which 24 (16%) have a role in transcriptional control and 28 (19%) are involved in signal transduction (Table S4). Fifty-seven of the 151 genes were (1) expressed in MKs based on a recent compendium of gene expression profiles in primary human hematopoietic cell types (Watkins et al., 2009) and (2) displayed increased expression in a recently published MK differentiation time series data set (Fuhrken et al., 2008). Eighteen of these 57 genes had known hematopoietic functions, and of the remaining 39 genes, zebrafish orthologs could be readily identified for 37 genes. Morpholino antisense oligonucleotides (MOs) were designed against eight of these genes, chosen randomly to provide an unbiased sampling, as well as against the TF *nfatc1*. *Nfact1* was included because even though NFATC1 had been implicated in transcriptional control in lymphocytes (Shaw et al., 1988), there were no prior data suggesting possible functions in megakaryopoiesis (see Table S5 for a summary of selected genes). MOs targeting these nine genes were injected into one-cell stage zebrafish embryos and assayed for their effect on two hematological traits: number of erythrocytes and number of thrombocytes. Remarkably, for all genes except *nfatc1*, injection of MOs caused a significant reduction in the number of circulating blood cells at 48 hr postfertilization (hpf) (Figure 5A) as confirmed by o-dianisidine staining for hemoglobin production. Whereas *emilin1* and *sufu* MOs had a mild effect on erythropoiesis, *ncor2*, *march2*, *smox*, *pttg1Ip*, *max*, and *pdzk1ip1l* MO injected embryos showed a profound reduction in the number of mature erythrocytes.

To further explore potential hematopoietic functions for the nine candidate genes, we examined *cd41* expression at 72 hpf in the caudal hematopoietic tissue (CHT) of MO injected fish using the *Tg*(*cd41*:*EGFP*) transgenic line (Lin et al., 2005) (Figures 5B and 5C; for pictures and cell counts in multiple embryos, see Table S6 and Figure S4). The CHT is widely recognized as the anatomic site of definitive hematopoiesis in zebrafish embryos, and *cd41*^low^ and *cd41*^high^ are considered to be the markers of hematopoietic stem cells (HSCs) and thrombocytes, respectively. For six genes (*march2*, *max*, *smox*, *pttg1lp*, *emilin1*, and *sufu*) MO depletion resulted in a severe decrease in the number of thrombocytes and HSCs. *Ncor2* depletion resulted in a mild phenotype. *Pdzk1ip1l* and *nfatc1* MO injected embryos showed no change in the number of *cd41* positive cells when compared to control embryos. Taken together, in vivo functional validation therefore demonstrated that genes adjacent to DNA sequences concurrently bound by GATA1, GATA2, RUNX1, FLI1, and SCL are highly enriched for previously unknown hematopoietic regulators.

### PDZK1IP1 Shares Transcriptional Enhancer Elements with the Essential Hematopoietic Stem Cell Regulator SCL

**Figure 6:**
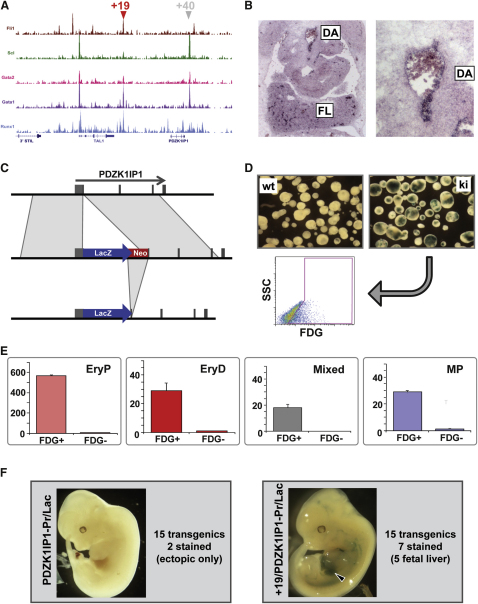
*PDZK1IP1* Shares Transcriptional Enhancer Elements with the Hematopoietic Master Regulator *SCL* (A) ChIP-Seq results (as for Figure 2) across the human *SCL*/*PDZK1IP1* gene locus with the location of the +19 and +40 enhancer elements indicated by arrowheads. (B) *Pdzk1ip1* is expressed in the fetal liver (FL) and dorsal aorta (DA) region of midgestation mouse embryos. Shown are the results from in situ hybridization experiments using transverse sections of day 12.5 mouse embryos with a magnified view of the dorsal aorta on the right hand side. (C) Targeting of a lacZ reporter gene into the 5′UTR of mouse *Pdzk1ip1* in ESCs. The neo selection marker was flanked by loxP sites and removed using Cre-mediated recombination. (D) *Pdzk1ip1* lacZ knockin ESCs express lacZ after differentiation into embryoid bodies. The top section shows day 6 embryoid bodies stained for lacZ using the chromogenic substrate X-Gal with wild-type (wt) cells on the left and *Pdzk1ip1* knockin cells (ki) on the right. The bottom part shows flow cytometry analysis using the fluorogenic lacZ substrate FDG with approximately 25% of *Pdzk1ip1* knockin cells expressing the lacZ reporter (FDG, x axis; Side Scatter SSC, y axis). (E) Hematopoietic colony forming activity is confined to *Pdzk1ip1* expressing cells in ESC differentiation assays. *Pdzk1ip1* expressing cells were purified by flow cytometry and analyzed for colony forming ability. Shown from left to right are colony numbers per 100,000 cells for primitive erythroid (EryP), definitive erythroid (EryD), mixed lineage (Mixed), and macrophage colonies (MP) (mean + SD). (F) The +19 enhancer drives expression to the fetal liver in transgenic mice when fused to the *Pdzk1ip1* promoter. Shown on the left is a representative E12.5 transgenic embryo carrying the *Pdzk1ip1* promoter fused to the lacZ reporter gene with no observable staining. Shown on the right is a representative transgenic embryo for the *Pdzk1ip1* promoter fused to the +19 enhancer with readily identifiable staining in the fetal liver (arrowhead). See also Figure S5.

Identification of a hematopoietic function in zebrafish for *pdzk1ip1I* was of particular interest because (1) its mammalian ortholog *PDZK1IP1* (also known as *MAP17*) encodes a membrane associated protein shown to modulate the levels of reactive oxygen species (Guijarro et al., 2007) that have recently emerged as important determinants of blood stem cell function (Tothova et al., 2007); (2) *PDZK1IP1* is situated immediately 3′ of the hematopoietic master regulator *SCL* (see Figure 6A); (3) this arrangement is conserved between mammals and zebrafish (Gottgens et al., 2002a); and (4) an erythroid enhancer regulating murine *Scl* expression is located 3′ of *PDZK1IP1* (+40 element; Figure 6A) (Delabesse et al., 2005). We previously argued that this *Scl* 3′ enhancer was the cause for close linkage between *Scl* and *Pdzk1ip1* during evolution because major rearrangements would have interfered with SCL expression (Ogilvy et al., 2007). However, the identification of a hematopoietic function for *pdzk1ip1l* suggests that the intimate linkage of SCL and PDZK1IP1 might be due to the fact they share regulatory elements to mediate their respective functions in hematopoietic cells. Interestingly, PDZK1IP1 is upregulated during megakaryocyte differentiation from CD34^+^ HPCs (Fuhrken et al., 2008) and we have previously observed that the *Scl* +19 enhancer targets expression to megakaryocytes (Silberstein et al., 2005).

To investigate the possibility of coregulation, we first determined expression of *Pdzk1ip1* in mouse embryos. As shown in Figure 6B, *Pdzk1ip1* was expressed in the midgestation mouse embryo both in the dorsal aorta (AGM region) and fetal liver in the hematopoietic domains that also express *Scl*. Of note, expression in dorsal aorta endothelium and hematopoietic clusters as well as a subset of fetal liver cells was highly reminiscent of the expression pattern previously seen in transgenic mouse embryos with the +19 enhancer fused to an *Scl*-promoter-lacZ reporter gene (Sanchez et al., 1999).

To examine hematopoietic expression of *Pdzk1ip1* further, we generated embryonic stem cells (ESCs) with a lacZ reporter gene knocked into the ATG start codon of *Pdzk1ip1* (Figures 6C, S5A, and S5B) and then used lacZ expression during ESC differentiation as a surrogate for *Pdzk1ip1* expression. ESCs were differentiated into embryoid bodies to produce hematopoietic cells that could be analyzed by flow cytometry and colony assays. At day 6 of differentiation, ∼25% of embryoid body cells derived from *Pdzk1ip1* knockin (KI) ESCs displayed lacZ staining (Figure 6D) with the vast majority of hematopoietic colony forming activity found in the lacZ expressing cells (Figure 6E). *Pdzk1ip1* expression therefore marks hematopoietic cells in both mouse embryos and differentiating ESCs within the known expression domain of *Scl*.

To investigate whether the regulatory elements previously shown to drive *Scl* expression can also act on the *Pdzk1ip1* promoter, we generated transgenic mouse embryos carrying the *Pdzk1ip1* promoter with and without the *Scl* +19 enhancer fused to a lacZ reporter gene. Of 15 transgenic embryos with the *Pdzk1ip1* promoter alone, only two showed expression (in the brain and limb buds, respectively). By contrast, 7 of 15 transgenic embryos for the promoter/enhancer construct showed staining, with five of seven embryos displaying strong staining in the fetal liver (Figure 6F) in a pattern highly reminiscent to what we observed previously for the +19 enhancer in conjunction with the *Scl* promoter (Sanchez et al., 1999). Of note, histological analysis demonstrated that LacZ expressing cells included fetal liver megakaryocytes (Figure S5C). These results demonstrate that a regulatory element previously thought to simply control expression of *Scl* can act in vivo to mediate expression from the *Pdzk1ip1* promoter. However, the Scl +19 enhancer was previously shown to interact with both Scl as well as heterologous promoters (Sanchez et al., 1999). The experiments presented here therefore do not directly prove that the +19 enhancer normally mediates specific Pdzk1ip1 regulation in vivo. Our results are nevertheless suggestive of a model whereby two very different proteins are maintained in close linkage throughout vertebrate evolution because they share key regulatory elements to sustain their expression in hematopoietic cells where they both perform important functions. Similar coregulatory relationships may be common throughout the genome, and thus an important consideration to be taken into account when utilizing ChIP-Seq studies for the reconstruction of regulatory networks.

## Discussion

Detailed molecular studies have shown individual TFs to play critical roles at various stages of MK maturation. For example, a hypomorph mouse model of *Gata1* displays reduced platelet numbers associated with deregulated MK proliferation and severely impaired cytoplasmic maturation (Shivdasani et al., 1997). The other TFs investigated in this study, GATA2, RUNX1, FLI1, and SCL, have also been previously shown to play important roles in MK differentiation (Growney et al., 2005; Hart et al., 2000; Huang et al., 2009b; Ichikawa et al., 2004; Mikkola et al., 2003; Song et al., 1999; Spyropoulos et al., 2000). Of note, these four factors also represent key players in regulatory network circuits operating in HSPCs (Gottgens et al., 2002b; Landry et al., 2009; Pimanda et al., 2007) consistent with the previously suggested notion of significant overlap between transcriptional control mechanisms in MKs and HSPCs (Huang and Cantor, 2009).

The five-factor ChIP-Seq data set generated made it feasible for us to perform a global analysis of combinatorial transcriptional control in a human primary myeloid cell type. Previous studies, which only examined individual promoter fragments, had revealed transcriptional cooperativity in MKs between RUNX1 and GATA1 (Elagib et al., 2003; Xu et al., 2006) as well as between FLI1 and GATA1 (Huang et al., 2009a). The latter was somewhat surprising given the well characterized antagonism between GATA1 and ETS family TFs during erythroid differentiation. Our genome-wide data sets support frequent co-occupancy of GATA1 and FLI1 in MK cells with a total of 1335 regions bound simultaneously by both factors. However, the vast majority (1094) of these are bound by additional factors and binding of GATA1 and FLI1 without any of the other factors was in fact the most underrepresented occupancy pattern. These observations suggest that activatory interactions between GATA1 and FLI1 may at least in part be mediated through the assembly of larger multiprotein complexes. Of particular relevance may be the observation that >700 regions are bound by GATA1 and FLI1 together with RUNX1. RUNX1 has been shown previously to interact at the protein level with both GATA1 and FLI1 (Huang et al., 2009a; Elagib et al., 2003). Moreover, RUNX1 expression is maintained in MKs whereas it is downregulated during erythroid differentiation (Elagib et al., 2003). Our combinatorial binding data are therefore consistent with a model where additional factors such as RUNX1 mediate transcriptional cooperation between GATA1 and FLI1 in MKs whereas antagonistic interactions may prevail in their absence as previously reported for the erythroid lineage.

Genes next to regions bound by all five factors were highly enriched for known regulators of MK differentiation and/or function. This observation prompted us to investigate the possibility that genes of unknown hematopoietic function with binding peaks for all five factors may also be enriched for the same functional categories. To provide biological insight, genome-scale hypothesis-generating screens such as ChIP-Seq experiments need to be coupled with meaningful assays for downstream functional validation. Here we took advantage of the zebrafish model because hematopoietic control mechanisms are highly conserved between zebrafish and human/mouse, yet only the zebrafish allows relatively high-throughput knockdown analysis in vivo in the context of a whole animal. Coupling the multifactor ChIP-Seq screen with zebrafish in vivo validation allowed us to identify eight regulators of thrombopoiesis and/or erythropoiesis. Of note, none of the MOs caused a lack of blood circulation and at the concentration used, with the exception of two (*smox* and *max*), MO knockdown did not affect wider aspects of morphology, underscoring the hematopoietic specificity of the uncovered phenotypes. Given that seven MOs affected both erythrocytes and thrombocytes, it is possible that at least some of those genes may operate at the level of immature progenitors or blood stem cells. Given the overlap of key transcriptional regulators in MKs and HSCs, potential functions in blood stem cells might perhaps not be too surprising but will require further fine dissection of knockdown phenotypes.

Recent ChIP-Seq studies of individual transcription factors in two different hematopoietic lineages suggested that binding patterns are largely lineage-specific (Heinz et al., 2010) with shared peaks in one study of Scl being as low as 6% (Palii et al., 2011). Comparison of the megakaryocyte data generated here with a recently published hematopoietic progenitor data set (Wilson et al., 2010a) confirmed the predominance of lineage-specific binding patterns, and in addition demonstrated that regions bound by multiple factors are also largely lineage-specific. This suggests that additional lineage-specific transcription factors may play a part in controlling cell-type specific accessibility of regulatory regions through interactions with chromatin modifying enzymes. The likely relevance of additional MK-specific transcription factors is further emphasized by our observation that the five factors studied here may only account for 30% of MK-specific expression. Additional important players within MK transcriptional programs are likely to include NF-E2, MEIS1, and E2A (Shivdasani, 1996; Hisa et al., 2004; Semerad et al., 2009). Our demonstration that PDZK1IP1 shares transcriptional regulatory elements with the blood stem cell regulator SCL has implications reaching beyond a better understanding of this particular gene locus. The notion that a given regulatory region can control multiple genes is well established, but past experimental analysis has largely focused on clusters of related genes such as the β-globin locus (Tolhuis et al., 2002) or tandem arrays of interleukin genes (Loots et al., 2000). Coregulation within these gene loci is likely to have arisen in parallel with local gene duplication events resulting in coordinated control of evolutionary and functionally related genes. By contrast, SCL and PDZK1IP1 encode unrelated proteins and it is likely that their original juxtaposition within vertebrate genomes was accidental. Our identification of PDZK1IP1 as a hematopoietic regulator together with the demonstration that SCL and PDZK1IP1 share transcriptional enhancer elements provides a rationale for the tight linkage between these two genes throughout vertebrate evolution. Moreover, our data illustrate the potential pitfalls of mapping transcription factor binding events to a single target gene when reconstructing regulatory networks. The +19 enhancer is approximately equidistant to the SCL and PDZK1IP1 promoters and evidently can control both. Transgenic in vivo analysis as performed here will be too time consuming and costly for global mapping of regulatory interactions between distal enhancers and the promoters of neighboring genes. However, further reductions in DNA sequencing costs may mean that chromatin-capture based methods providing a genome-wide view of promoter/enhancer interactions (Fullwood et al., 2009) will eventually perform this integral part of regulatory network reconstruction.

Previous multi-TF ChIP-Seq studies have revealed layers of information that can only be obtained from integrated analysis of multiple factors. For example, computational analysis of a 12-factor ChIP-Seq data set from mouse ESCs suggested that genome-scale analysis of combinatorial TF occupancy has the potential to predict absolute and differential gene expression (Ouyang et al., 2009). A five-factor ChIP-Seq study in an erythroid model cell line revealed that multi-TF complex binding often marks sites of long-range genomic interactions (Soler et al., 2010). Here we have shown that multifactor ChIP-Seq surveys coupled to high-throughput in vivo functional screening provide a powerful strategy toward isolating key regulators of cellular phenotypes. Future detailed functional analyses of the eight hematopoietic regulators identified here, has the potential to integrate new regulatory pathways into an emerging framework of blood development.

## Experimental Procedures

### Megakaryocyte Culture

Cord blood was obtained after informed consent under a protocol approved by the National Research Ethics Service. CD34-positive cells (≥98%) isolated by magnetic cell sorting (Myltenyi Biotec), were seeded at 1 × 10^5^ cells/ml in CellGro SCGM medium (CellGenix) with 100 ng/ml human TPO (CellGenix) and 10 ng/ml IL-1β (Miltenyi Biotec) and incubated for 10 days. Flow cytometry was performed on a CyAn ADP 9 color (Beckman Coulter) as described (Macaulay et al., 2007) using the following antibodies: CD11c^PE-Cy5^ or ^V450^ (clone B-ly6; Becton Dickinson [BD]), CD14^PB^ (clone M5E2; BD), CD15^APC^ (clone HI98; BD) or ^V450^ (clone MMA; BD), CD34^PE^ (clone 581; Beckman Coulter), CD41^APC^ (clone HIP8; BD), CD42a^F^ (clone ALMA.16; BD), CD66^PE^ (clone B6.2; BD), and IgG^APC^ (clone MOPC-21; BD).

### Chromatin Immunoprecipitation

ChIP assays were performed as previously described (Forsberg et al., 2000) with 9.4–12.6 × 10^6^ cells and anti-GATA1 (ab11963, Abcam), anti-GATA2 (clone H-116, Santa Cruz), anti-RUNX1 (ab23980, Abcam), anti-FLI1 (ab15289, Abcam), anti-TAL1 antibody (clone C-21, Santa Cruz) and nonspecific Rabbit IgG (I5006; Sigma Aldrich). Samples were sequenced using the Illumina GII Genome Analyzer. Sequence data have been submitted to http://www.ncbi.nlm.nih.gov/geo (GEO record GSE24674).

### ChIP-Seq Analysis

Three peak finding programs (Peakseq [Rozowsky et al., 2009], Findpeaks [Fejes et al., 2008] and model-based analysis of ChIP-Seq [MACS] [Zhang et al., 2008]) were used, based on previous observations that there is no single algorithm of choice when calling peak regions for different TFs (Laajala et al., 2009). De novo motif discovery was performed using MEME (Bailey and Elkan, 1994) or bioprospector (Liu et al., 2001) and motifs were compared with the JASPAR_CORE database (Bryne et al., 2008). Matches to consensus sequences were determined as described using TFBSSearch (Chapman et al., 2004). Peaks in promoters and introns were allocated to that gene and the remainder of peaks to the nearest 3′ and 5′ genes in a 100 kb region. Intersects of gene lists were generated using Galaxy (Goecks et al., 2010), gene ontology terms were analyzed using GO Term Mapper (Lewis-Sigler Institute for Integrative Genomics at Princeton University), and gene set enrichment analysis was performed using the GSEA software (Subramanian et al., 2005).

### Zebrafish Knockdown

General maintenance, collection, and staging were carried out according to the Zebrafish Book (Westerfield, 1994). Antisense MOs (Gene Tools) were designed complementary to the 5′ sequence near the start of translation (*max* and *sufu*), or at splice junctions (*ncor2, march2, smox*, *nfatc1*, *pttg1lp*, *emilin1*, and *pdzk1ip1l*) (Table S7). An aliquot of 0.8 nl morpholino-containing solution was injected in zebrafish embryos at the one- to two-cell stage. For *march2* and *pdzk1ip1l* a MO concentration of 3 μg/μl and for *ncor2, smox*, *nfatc1*, *pttg1lp*, *max*, *emilin1*, and *sufu* a MO concentration of 6 μg/μl was used. Efficiency of splice-site MOs was determined by RT-PCR (Table S7). Total RNA was isolated from control and splice MO injected embryos using the RNeasy Mini Kit (QIAGEN) and cDNA synthesized using SuperScript III (Invitrogen). Staining of hemoglobin by o-dianisidine was performed as described (Detrich et al., 1995). Photomicrographs were taken with a Zeiss camera AxioCam HRC attached to a LeicaMZ16 FA dissecting microscope (Leica Microsystems).

### *Pdzk1ip1* KI Mouse ESCs

To generate *Pdzk1ip1* KI ESCs a targeting vector was generated by bacterial recombineering (Liu et al., 2003) where 131bp of exon 1 of *Pdzk1ip1* (containing the ATG site) were substituted by a LacZ reporter gene and a LoxP-PGK-Neo-LoxP cassette. Targeting was determined by Southern blot using 5′ and 3′ probes outside the targeting vector. Targeted cells were transiently transfected with PGK-Cre for deletion of the Neo cassette (Figure S5). *Pdzk1ip1* KI ES were differentiated into embryoid bodies as described (Keller et al., 1993) and stained with X-gal as described (Smith et al., 2008). For sorting, embryoid bodies were disrupted at day 6 of differentiation and stained with Fluorescein di(β-D-galactopyranoside) (FDG) (Sanchez et al., 1999). Cells were plated in methocult (Stem Cell Technologies) with appropriate cytokines (Peprotech, R&D) and scored after 7 days.

### Transgenic Mice and In Situ Hybridization

*Pdzk1ip1*-Pr/Lac and +19/*Pdzk1ip1*-Pr/Lac transgenic constructs were generated by inserting a 4.5 kb fragment containing the *Pdzk1ip1* promoter and a 640 bp fragment containing the +19 enhancer into the pGLac vector. Methods for transgenic mice generation and in situ hybridization are described elsewhere (Wilson et al., 2010b).

## Figures and Tables

**Table 1:** GREAT Analysis Shows Highly Significant Overrepresentation of Hematopoietic Phenotypes and MK Gene Expression Patterns for the Regions Bound by All Five TFs

Enriched Term	Raw p value	FDR q value
Abnormal hematopoietic system physiology	3.5 E-07	2.0 E-03
Hematopoietic system phenotype	6.8 E-07	2.0 E-03
Anemia	1.5 E-06	2.9 E-03
Abnormal hematopoietic system/development	2.1 E-06	3.0 E-03
Abnormal hematopoiesis	3.5 E-06	4.0 E-03
Anemia	4.4 E-06	4.2 E-03
Abnormal blood cell morphology/development	6.6 E-06	5.5 E-03
Abnormal immune system morphology	1.2 E-06	8.6 E-03
Abnormal leukocyte cell number	2.8 E-05	1.5 E-02
Abnormal leukocyte morphology	4.3 E-05	2.1 E-02
Abnormal immune system cell morphology	5.1 E-05	2.1 E-02
Abnormal mononuclear leukocyte cell morphology	7.2 E-05	2.8 E-02
Decreased platelet cell number	1.6 E-04	4.6 E-02
MsigDB gene sets perturbation		
Genes upregulated in AML samples of the FAB class M7	3.1 E-06	2.8 E-03
Genes essential to the development of megakaryocytes, as expressed in normal cells and essential thrombocythemic cells	1.0 E-05	4.6 E-03

FDR, false discovery rate; GREAT, genomic regions enrichment of annotations tool; TFs, transcription factors.

## Accession Numbers

Sequence data have been submitted to the NCBI Gene Expression Omnibus database (accession number GSE24674).

## Supplemental Information

Document S1. Supplemental Experimental Procedures, Supplemental References, Five Figures, and Four Tables

Table S1. Peak Coordinates for the 31 Binding Patterns of GATA1, GATA2, RUNX1, FLI1, and SCL, Related to Figure 3

Table S2. Candidate Target Genes for the 31 Binding Patterns of GATA1, GATA2, RUNX1, FLI1, and SCL, Related to Figure 3

Table S3. Gene Set Enrichment Analysis for the 31 Binding Patterns of GATA1, GATA2, RUNX1, FLI1, and SCL, Related to Figure 4

## References

[bib1] Adolfsson J., Mansson R., Buza-Vidas N., Hultquist A., Liuba K., Jensen C.T., Bryder D., Yang L., Borge O.J., Thoren L.A. (2005). Identification of Flt3^+^ lympho-myeloid stem cells lacking erythro-megakaryocytic potential a revised road map for adult blood lineage commitment. Cell.

[bib2] Anderson K.P., Crable S.C., Lingrel J.B. (1998). Multiple proteins binding to a GATA-E box-GATA motif regulate the erythroid Kruppel-like factor (EKLF) gene. J. Biol. Chem..

[bib3] Bailey T.L., Elkan C. (1994). Fitting a mixture model by expectation maximization to discover motifs in biopolymers. Proc. Int. Conf. Intell. Syst. Mol. Biol..

[bib4] Boyle A.P., Davis S., Shulha H.P., Meltzer P., Margulies E.H., Weng Z., Furey T.S., Crawford G.E. (2008). High-resolution mapping and characterization of open chromatin across the genome. Cell.

[bib5] Bryne J.C., Valen E., Tang M.-H.E., Marstrand T., Winther O., da Piedade I., Krogh A., Lenhard B., Sandelin A. (2008). JASPAR, the open access database of transcription factor-binding profiles: new content and tools in the 2008 update. Nucleic Acids Res..

[bib6] Chapman M.A., Donaldson I.J., Gilbert J., Grafham D., Rogers J., Green A.R., Gottgens B. (2004). Analysis of multiple genomic sequence alignments: a web resource, online tools, and lessons learned from analysis of mammalian SCL loci. Genome Res..

[bib7] Davidson E.H. (2001). Genomic Regulatory Systems: Development and Evolution.

[bib8] Davidson E.H. (2010). Emerging properties of animal gene regulatory networks. Nature.

[bib9] Delabesse E., Ogilvy S., Chapman M.A., Piltz S.G., Gottgens B., Green A.R. (2005). Transcriptional regulation of the SCL locus: identification of an enhancer that targets the primitive erythroid lineage in vivo. Mol. Cell. Biol..

[bib10] Detrich H.W., Kieran M.W., Chan F.Y., Barone L.M., Yee K., Rundstadler J.A., Pratt S., Ransom D., Zon L.I. (1995). Intraembryonic hematopoietic cell migration during vertebrate development. Proc. Natl. Acad. Sci. USA.

[bib11] Elagib K.E., Racke F.K., Mogass M., Khetawat R., Delehanty L.L., Goldfarb A.N. (2003). RUNX1 and GATA-1 coexpression and cooperation in megakaryocytic differentiation. Blood.

[bib12] Fejes A.P., Robertson G., Bilenky M., Varhol R., Bainbridge M., Jones S.J. (2008). FindPeaks 3.1: a tool for identifying areas of enrichment from massively parallel short-read sequencing technology. Bioinformatics.

[bib13] Forsberg E.C., Downs K.M., Bresnick E.H. (2000). Direct interaction of NF-E2 with hypersensitive site 2 of the beta-globin locus control region in living cells. Blood.

[bib14] Fuhrken P.G., Chen C., Apostolidis P.A., Wang M., Miller W.M., Papoutsakis E.T. (2008). Gene Ontology-driven transcriptional analysis of CD34^+^ cell-initiated megakaryocytic cultures identifies new transcriptional regulators of megakaryopoiesis. Physiol. Genomics.

[bib15] Fujiwara T., O'Geen H., Keles S., Blahnik K., Linnemann A.K., Kang Y.A., Choi K., Farnham P.J., Bresnick E.H. (2009). Discovering hematopoietic mechanisms through genome-wide analysis of GATA factor chromatin occupancy. Mol. Cell.

[bib16] Fullwood M.J., Liu M.H., Pan Y.F., Liu J., Xu H., Mohamed Y.B., Orlov Y.L., Velkov S., Ho A., Mei P.H. (2009). An oestrogen-receptor-alpha-bound human chromatin interactome. Nature.

[bib17] Goecks J., Nekrutenko A., Taylor J. (2010). Galaxy: a comprehensive approach for supporting accessible, reproducible, and transparent computational research in the life sciences. Genome Biol..

[bib18] Gottgens B., Barton L.M., Chapman M.A., Sinclair A.M., Knudsen B., Grafham D., Gilbert J.G., Rogers J., Bentley D.R., Green A.R. (2002). Transcriptional regulation of the stem cell leukemia gene (SCL)—comparative analysis of five vertebrate SCL loci. Genome Res..

[bib19] Gottgens B., Nastos A., Kinston S., Piltz S., Delabesse E.C., Stanley M., Sanchez M.J., Ciau-Uitz A., Patient R., Green A.R. (2002). Establishing the transcriptional programme for blood: the SCL stem cell enhancer is regulated by a multiprotein complex containing Ets and GATA factors. EMBO J..

[bib20] Growney J.D., Shigematsu H., Li Z., Lee B.H., Adelsperger J., Rowan R., Curley D.P., Kutok J.L., Akashi K., Williams I.R. (2005). Loss of Runx1 perturbs adult hematopoiesis and is associated with a myeloproliferative phenotype. Blood.

[bib21] Guijarro M.V., Leal J.F., Blanco-Aparicio C., Alonso S., Fominaya J., Lleonart M., Castellvi J., Cajal S., Carnero A. (2007). MAP17 enhances the malignant behavior of tumor cells through ROS increase. Carcinogenesis.

[bib22] Hart A., Melet F., Grossfeld P., Chien K., Jones C., Tunnacliffe A., Favier R., Bernstein A. (2000). Fli-1 is required for murine vascular and megakaryocytic development and is hemizygously deleted in patients with thrombocytopenia. Immunity.

[bib23] Heinz S., Benner C., Spann N., Bertolino E., Lin Y.C., Laslo P., Cheng J.X., Murre C., Singh H., Glass C.K. (2010). Simple combinations of lineage-determining transcription factors prime cis-regulatory elements required for macrophage and B cell identities. Mol. Cell.

[bib24] Hisa T., Spence S.E., Rachel R.A., Fujita M., Nakamura T., Ward J.M., Devor-Henneman D.E., Saiki Y., Kutsuna H., Tessarollo L. (2004). Hematopoietic, angiogenic and eye defects in Meis1 mutant animals. EMBO J..

[bib25] Huang H., Cantor A.B. (2009). Common features of megakaryocytes and hematopoietic stem cells: what's the connection?. J. Cell. Biochem..

[bib26] Huang H., Yu M., Akie T.E., Moran T.B., Woo A.J., Tu N., Waldon Z., Lin Y.Y., Steen H., Cantor A.B. (2009). Differentiation-dependent interactions between RUNX-1 and FLI-1 during megakaryocyte development. Mol. Cell. Biol..

[bib27] Huang Z., Dore L.C., Li Z., Orkin S.H., Feng G., Lin S., Crispino J.D. (2009). GATA-2 reinforces megakaryocyte development in the absence of GATA-1. Mol. Cell. Biol..

[bib28] Ichikawa M., Asai T., Saito T., Seo S., Yamazaki I., Yamagata T., Mitani K., Chiba S., Ogawa S., Kurokawa M. (2004). AML-1 is required for megakaryocytic maturation and lymphocytic differentiation, but not for maintenance of hematopoietic stem cells in adult hematopoiesis. Nat. Med..

[bib29] Kassouf M.T., Hughes J.R., Taylor S., McGowan S.J., Soneji S., Green A.L., Vyas P., Porcher C. (2010). Genome-wide identification of TAL1's functional targets: insights into its mechanisms of action in primary erythroid cells. Genome Res..

[bib30] Keller G., Kennedy M., Papayannopoulou T., Wiles M.V. (1993). Hematopoietic commitment during embryonic stem cell differentiation in culture. Mol. Cell. Biol..

[bib31] Laajala T.D., Raghav S., Tuomela S., Lahesmaa R., Aittokallio T., Elo L.L. (2009). A practical comparison of methods for detecting transcription factor binding sites in ChIP-seq experiments. BMC Genomics.

[bib32] Landry J.R., Bonadies N., Kinston S., Knezevic K., Wilson N.K., Oram S.H., Janes M., Piltz S., Hammett M., Carter J. (2009). Expression of the leukemia oncogene Lmo2 is controlled by an array of tissue-specific elements dispersed over 100 kb and bound by Tal1/Lmo2, Ets, and Gata factors. Blood.

[bib33] Lin H.F., Traver D., Zhu H., Dooley K., Paw B.H., Zon L.I., Handin R.I. (2005). Analysis of thrombocyte development in CD41-GFP transgenic zebrafish. Blood.

[bib34] Lin Y.C., Jhunjhunwala S., Benner C., Heinz S., Welinder E., Mansson R., Sigvardsson M., Hagman J., Espinoza C.A., Dutkowski J. (2010). A global network of transcription factors, involving E2A, EBF1 and Foxo1, that orchestrates B cell fate. Nat. Immunol..

[bib35] Liu X., Brutlag D.L., Liu J.S. (2001). BioProspector: discovering conserved DNA motifs in upstream regulatory regions of co-expressed genes. Pac. Symp. Biocomput..

[bib36] Liu P., Jenkins N.A., Copeland N.G. (2003). A highly efficient recombineering-based method for generating conditional knockout mutations. Genome Res..

[bib37] Loots G.G., Locksley R.M., Blankespoor C.M., Wang Z.E., Miller W., Rubin E.M., Frazer K.A. (2000). Identification of a coordinate regulator of interleukins 4, 13, and 5 by cross-species sequence comparisons. Science.

[bib38] Macaulay I.C., Tijssen M.R., Thijssen-Timmer D.C., Gusnanto A., Steward M., Burns P., Langford C.F., Ellis P.D., Dudbridge F., Zwaginga J.J. (2007). Comparative gene expression profiling of in vitro differentiated megakaryocytes and erythroblasts identifies novel activatory and inhibitory platelet membrane proteins. Blood.

[bib39] McDaniell R., Lee B.K., Song L., Liu Z., Boyle A.P., Erdos M.R., Scott L.J., Morken M.A., Kucera K.S., Battenhouse A. (2010). Heritable individual-specific and allele-specific chromatin signatures in humans. Science.

[bib40] McLean C.Y., Bristor D., Hiller M., Clarke S.L., Schaar B.T., Lowe C.B., Wenger A.M., Bejerano G. (2010). GREAT improves functional interpretation of cis-regulatory regions. Nat. Biotechnol..

[bib41] Mikkola H.K., Klintman J., Yang H., Hock H., Schlaeger T.M., Fujiwara Y., Orkin S.H. (2003). Haematopoietic stem cells retain long-term repopulating activity and multipotency in the absence of stem-cell leukaemia SCL/tal-1 gene. Nature.

[bib42] Minami T., Tachibana K., Imanishi T., Doi T. (1998). Both Ets-1 and GATA-1 are essential for positive regulation of platelet factor 4 gene expression. Eur. J. Biochem..

[bib43] Ogilvy S., Ferreira R., Piltz S.G., Bowen J.M., Gottgens B., Green A.R. (2007). The SCL +40 enhancer targets the midbrain together with primitive and definitive hematopoiesis and is regulated by SCL and GATA proteins. Mol. Cell. Biol..

[bib44] Orkin S.H., Zon L.I. (2008). Hematopoiesis: an evolving paradigm for stem cell biology. Cell.

[bib45] Ouyang Z., Zhou Q., Wong W.H. (2009). ChIP-Seq of transcription factors predicts absolute and differential gene expression in embryonic stem cells. Proc. Natl. Acad. Sci. USA.

[bib46] Palii C.G., Perez-Iratxeta C., Yao Z., Cao Y., Dai F., Davison J., Atkins H., Allan D., Dilworth F.J., Gentleman R. (2011). Differential genomic targeting of the transcription factor TAL1 in alternate haematopoietic lineages. EMBO J..

[bib47] Pencovich N., Jaschek R., Tanay A., Groner Y. (2011). Dynamic combinatorial interactions of RUNX1 and cooperating partners regulates megakaryocytic differentiation in cell line models. Blood.

[bib48] Pimanda J.E., Ottersbach K., Knezevic K., Kinston S., Chan W.Y., Wilson N.K., Landry J.R., Wood A.D., Kolb-Kokocinski A., Green A.R. (2007). Gata2, Fli1, and Scl form a recursively wired gene-regulatory circuit during early hematopoietic development. Proc. Natl. Acad. Sci. USA.

[bib49] Roy S., Ernst J., Kharchenko P.V., Kheradpour P., Negre N., Eaton M.L., Landolin J.M., Bristow C.A., Ma L., Lin M.F. (2010). Identification of functional elements and regulatory circuits by Drosophila modENCODE. Science.

[bib50] Rozowsky J., Euskirchen G., Auerbach R.K., Zhang Z.D., Gibson T., Bjornson R., Carriero N., Snyder M., Gerstein M.B. (2009). PeakSeq enables systematic scoring of ChIP-seq experiments relative to controls. Nat. Biotechnol..

[bib51] Sanchez M., Gottgens B., Sinclair A.M., Stanley M., Begley C.G., Hunter S., Green A.R. (1999). An SCL 3′ enhancer targets developing endothelium together with embryonic and adult haematopoietic progenitors. Development.

[bib52] Semerad C.L., Mercer E.M., Inlay M.A., Weissman I.L., Murre C. (2009). E2A proteins maintain the hematopoietic stem cell pool and promote the maturation of myelolymphoid and myeloerythroid progenitors. Proc. Natl. Acad. Sci. USA.

[bib53] Shaw J.P., Utz P.J., Durand D.B., Toole J.J., Emmel E.A., Crabtree G.R. (1988). Identification of a putative regulator of early T cell activation genes. Science.

[bib54] Shivdasani R.A. (1996). The role of transcription factor NF-E2 in megakaryocyte maturation and platelet production. Stem Cells.

[bib55] Shivdasani R.A., Fujiwara Y., McDevitt M.A., Orkin S.H. (1997). A lineage-selective knockout establishes the critical role of transcription factor GATA-1 in megakaryocyte growth and platelet development. EMBO J..

[bib56] Silberstein L., Sanchez M.J., Socolovsky M., Liu Y., Hoffman G., Kinston S., Piltz S., Bowen M., Gambardella L., Green A.R. (2005). Transgenic analysis of the stem cell leukemia +19 stem cell enhancer in adult and embryonic hematopoietic and endothelial cells. Stem Cells.

[bib57] Smith A.M., Sanchez M.J., Follows G.A., Kinston S., Donaldson I.J., Green A.R., Gottgens B. (2008). A novel mode of enhancer evolution: the Tal1 stem cell enhancer recruited a MIR element to specifically boost its activity. Genome Res..

[bib58] Soler E., Andrieu-Soler C., de Boer E., Bryne J.C., Thongjuea S., Stadhouders R., Palstra R.J., Stevens M., Kockx C., van Ijcken W. (2010). The genome-wide dynamics of the binding of Ldb1 complexes during erythroid differentiation. Genes Dev..

[bib59] Song W.J., Sullivan M.G., Legare R.D., Hutchings S., Tan X., Kufrin D., Ratajczak J., Resende I.C., Haworth C., Hock R. (1999). Haploinsufficiency of CBFA2 causes familial thrombocytopenia with propensity to develop acute myelogenous leukaemia. Nat. Genet..

[bib60] Spyropoulos D.D., Pharr P.N., Lavenburg K.R., Jackers P., Papas T.S., Ogawa M., Watson D.K. (2000). Hemorrhage, impaired hematopoiesis, and lethality in mouse embryos carrying a targeted disruption of the Fli1 transcription factor. Mol. Cell. Biol..

[bib61] Subramanian A., Tamayo P., Mootha V.K., Mukherjee S., Ebert B.L., Gillette M.A., Paulovich A., Pomeroy S.L., Golub T.R., Lander E.S. (2005). Gene set enrichment analysis: a knowledge-based approach for interpreting genome-wide expression profiles. Proc. Natl. Acad. Sci. USA.

[bib62] Tolhuis B., Palstra R.J., Splinter E., Grosveld F., de Laat W. (2002). Looping and interaction between hypersensitive sites in the active beta-globin locus. Mol. Cell.

[bib63] Tothova Z., Kollipara R., Huntly B.J., Lee B.H., Castrillon D.H., Cullen D.E., McDowell E.P., Lazo-Kallanian S., Williams I.R., Sears C. (2007). FoxOs are critical mediators of hematopoietic stem cell resistance to physiologic oxidative stress. Cell.

[bib64] Wadman I.A., Osada H., Grutz G.G., Agulnick A.D., Westphal H., Forster A., Rabbitts T.H. (1997). The LIM-only protein Lmo2 is a bridging molecule assembling an erythroid, DNA-binding complex which includes the TAL1, E47, GATA-1 and Ldb1/NLI proteins. EMBO J..

[bib65] Watkins N.A., Gusnanto A., de Bono B., De S., Miranda-Saavedra D., Hardie D.L., Angenent W.G., Attwood A.P., Ellis P.D., Erber W. (2009). A HaemAtlas: characterizing gene expression in differentiated human blood cells. Blood.

[bib66] Westerfield M. (1994). The Zebrafish Book. A Guide for the Laboratory Use of Zebrafish (Dania rerio).

[bib67] Wilson N.K., Foster S.D., Wang X., Knezevic K., Schutte J., Kaimakis P., Chilarska P.M., Kinston S., Ouwehand W.H., Dzierzak E. (2010). Combinatorial transcriptional control in blood stem/progenitor cells: genome-wide analysis of ten major transcriptional regulators. Cell Stem Cell.

[bib68] Wilson N.K., Timms R.T., Kinston S.J., Cheng Y.H., Oram S.H., Landry J.R., Mullender J., Ottersbach K., Gottgens B. (2010). Gfi1 expression is controlled by five distinct regulatory regions spread over 100 kilobases, with Scl/Tal1, Gata2, PU.1, Erg, Meis1, and Runx1 acting as upstream regulators in early hematopoietic cells. Mol. Cell. Biol..

[bib69] Xu G., Kanezaki R., Toki T., Watanabe S., Takahashi Y., Terui K., Kitabayashi I., Ito E. (2006). Physical association of the patient-specific GATA1 mutants with RUNX1 in acute megakaryoblastic leukemia accompanying Down syndrome. Leukemia.

[bib70] Yamaguchi Y., Zon L.I., Ackerman S.J., Yamamoto M., Suda T. (1998). Forced GATA-1 expression in the murine myeloid cell line M1: induction of c-Mpl expression and megakaryocytic/erythroid differentiation. Blood.

[bib71] Zhang Y., Liu T., Meyer C.A., Eeckhoute J., Johnson D.S., Bernstein B.E., Nusbaum C., Myers R.M., Brown M., Li W. (2008). Model-based analysis of ChIP-Seq (MACS). Genome Biol..

