# Supplementary material for: Genome-wide Analysis of Simultaneous GATA1/2, RUNX1, FLI1, and SCL Binding in Megakaryocytes Identifies Hematopoietic Regulators
Source: Dev Cell. 2011 May 17;20(5):597–609. doi: 10.1016/j.devcel.2011.04.008 (PMC3145975; doi:10.1016/j.devcel.2011.04.008)
Supplement: Document S1. Supplemental Experimental Procedures, Supplemental References, Five Figures, and Four Tables [file mmc1.pdf]

**Developmental Cell, Volume *20***

## Supplemental Information

# Genome-Wide Analysis of Simultaneous GATA1/2, RUNX1, FLI1, and SCL Binding in Megakaryocytes Identifies Hematopoietic Regulators

**Marloes R. Tijssen, Ana Cvejic, Anagha Joshi, Rebecca L. Hannah, Rita Ferreira, Ariel Forrai, Dana C. Bellissimo, S. Helen Oram, Peter A. Smethurst, Nicola K. Wilson, Xiaonan Wang, Katrin Ottersbach, Derek L. Stemple, Anthony R. Green, Willem H. Ouwehand, and Berthold Göttgens**

## Supplemental Experimental Procedures

### Histone modification ChIP-Sequencing

Megakaryocytes were cultured as described for TF ChIPs. ChIP assays were performed using 33.4 million cells using anti-trimethyl-Histone H3 (Lys4; clone MC315, Millipore), Anti-acetyl-Histone H3 (Millipore), and Anti-trimethyl-Histone H3 (Lys27, Millipore). Patterns for H3K27me3, H3K4me3 and H3K9ac around the HOXA loci show active genes on the right hand side with repressive marks on the left. The homo sapiens promoter database (Cold Spring Harbor Laboratory) was used to depict the histone marks on all promoters.

### Validation of the ChIP material by Real-time PCR

Enrichment for the GATA1 ChIP was determined with the following primers: SCL –16kb forward; 5’-TGTAAGTGGCAGTGGTGATGTCT-3’, reverse; 5’-AACAGAACACACTTGAAGTGGCA-3’. SCL +40kb forward; 5’-TGGGAATGAGCGATAAGGAT-3’, reverse; 5’-TTACAGCCCTTCACCCTCAC-3’. Enrichment for all other ChIPs was quantified with the following primers: RUNX1 +23kb forward; 5’-CCTGTGGTTTTCTCGCTCTC-3’, reverse; 5’-ATGCTGACAGCCTCAGATGG-3’. RUNX1 +31kb forward; 5’-AAAGCCACAAAAAGAGATCTGG-3’, reverse; 5’-AGCACCTGCCAGAAGACATC-3’.

**Peak calling**

Peak calling programs and parameters used for the different TFs were as follows. GATA1: overlap of all three programs with Findpeaks – FDR <0.01 called, MACS – mfold=16 --tsize=35 --bw=100 --pvalue=1e-9 --gsize=2200000000 –nolambda, Peakseq – p=0.01. GATA2: MACS – mfold=16 --tsize=35 --bw=100 --pvalue=1e-9 --gsize=2200000000 –nolambda. RUNX1: overlap of all three programs with Findpeaks – FDR 0 called, MACS – mfold=16 --tsize=35 --bw=100 --pvalue=1e-15 --gsize=2200000000 –nolambda, Peakseq – p=1e-7. FLI1: Findpeaks – default setting for ChIPseq analysis, peaks with FDR < 0.05 called. SCL: Peakseq – standard settings (p=0.05). When the overlap of three algorithms was used, MACS peak coordinates were used as reference for further analysis.

**Generation of *Pdzk1ip1* targeted ES cell lines**

To inactivate *Pdzk1ip1* a targeting vector was generated by bacterial recombineering which was designed to delete 131bp of exon 1 of *Pdzk1ip1* (containing the ATG site) and insert a LacZ reporter gene and a LoxP-PGK-Neo-LoxP cassette (Liu et al., 2003). The targeting vector was used for homologous recombination in AB2.2 ES cells. Targeted cells were transiently transfected with PGK-Cre plasmid for deletion of the Neo cassette.

## Supplemental References


Liu,P., Jenkins,N.A., and Copeland,N.G. (2003). A highly efficient recombineering-based method for generating conditional knockout mutations. Genome Res. 13, 476-484.

Matsumura,G. and Sasaki,K. (1989). Megakaryocytes in the yolk sac, liver and bone marrow of the mouse: a cytometrical analysis by semithin light microscopy. J. Anat. 167, 181-187.

Ye,T., Krebs,A.R., Choukrallah,M.A., Keime,C., Plewniak,F., Davidson,I., and Tora,L. (2010). seqMINER: an integrated ChIP-seq data interpretation platform. Nucleic Acids Res.

A

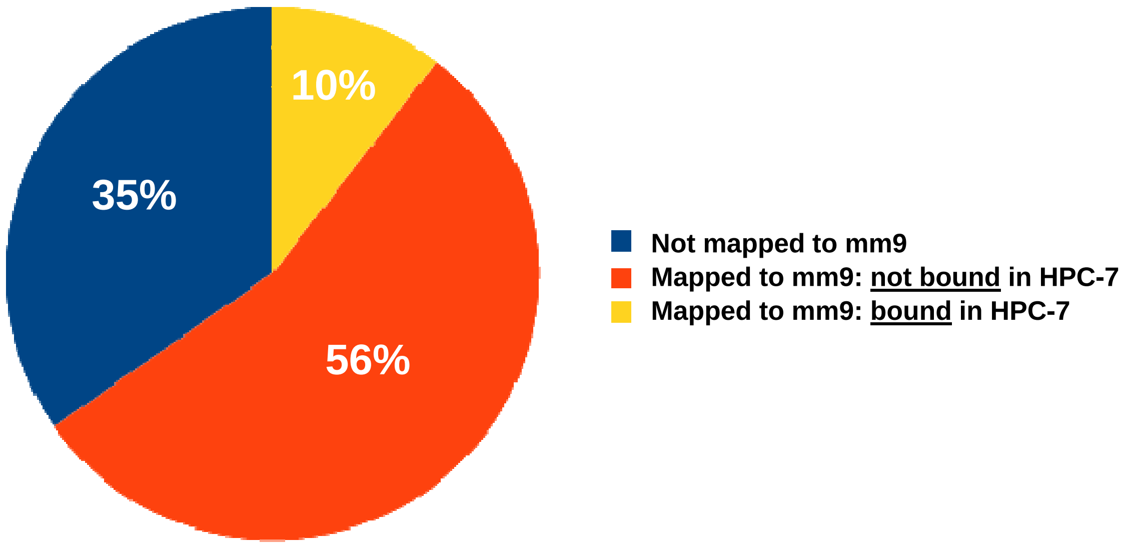


B

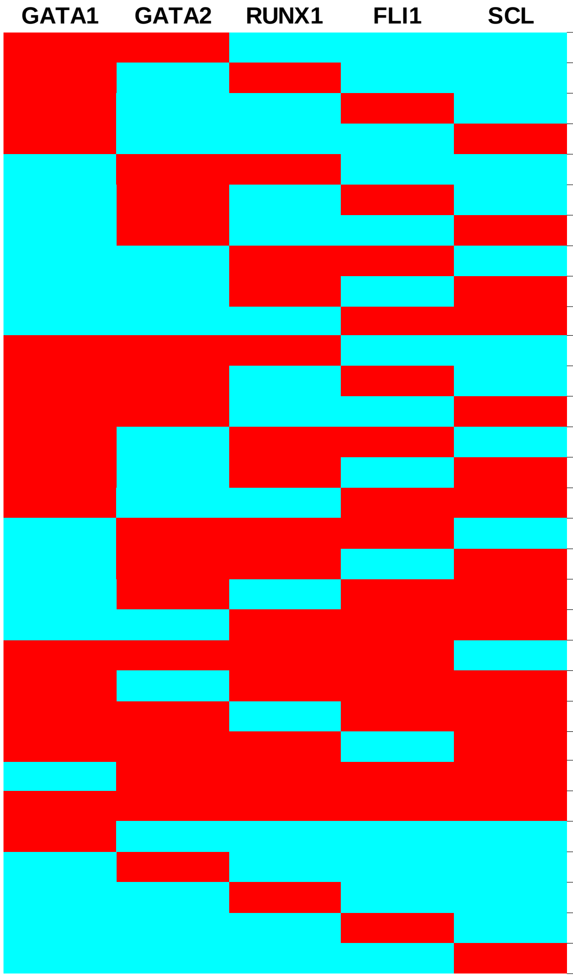


| Peaks | Mapped to mm9 (%) | Mapped to mm9: not bound in HPC-7 | Mapped to mm9: bound in HPC-7 (%) |
|---|---|---|---|
| 224 | 17 (8) | 17 | 0 (0) |
| 419 | 216 (52) | 208 | 8 (4) |
| 241 | 157 (65) | 147 | 10 (6) |
| 371 | 249 (67) | 222 | 27 (11) |
| 44 | 33 (75) | 33 | 0 (0) |
| 324 | 60 (19) | 60 | 0 (0) |
| 18 | 7 (39) | 7 | 0 (0) |
| 1586 | 1336 (84) | 1253 | 83 (6) |
| 83 | 54 (65) | 49 | 5 (9) |
| 335 | 234 (70) | 206 | 28 (12) |
| 66 | 55 (83) | 46 | 9 (16) |
| 72 | 28 (39) | 27 | 1 (4) |
| 64 | 54 (84) | 41 | 13 (24) |
| 221 | 155 (70) | 145 | 10 (6) |
| 140 | 100 (71) | 86 | 14 (14) |
| 264 | 188 (71) | 156 | 32 (17) |
| 34 | 19 (56) | 18 | 1 (5) |
| 2 | 2 (100) | 2 | 0 (0) |
| 26 | 8 (31) | 8 | 0 (0) |
| 133 | 105 (79) | 92 | 13 (12) |
| 104 | 32 (31) | 26 | 6 (19) |
| 235 | 174 (74) | 174 | 0 (0) |
| 54 | 38 (70) | 30 | 8 (21) |
| 46 | 33 (72) | 25 | 8 (24) |
| 2 | 1 (50) | 1 | 0 (0) |
| 144 | 103 (72) | 80 | 23 (22) |
| 1821 | 1069 (59) | 997 | 72 (7) |
| 858 | 487 (57) | 462 | 25 (5) |
| 3644 | 2716 (75) | 2594 | 122 (4) |
| 4539 | 3095 (68) | 2340 | 755 (24) |
| 1149 | 707 (62) | 665 | 42 (6) |

# Tijssen et al Supplemental Figure 1

**Figure S1. Comparison of the Megakaryocyte Data with Hematopoietic Progenitor Data, related to Figure 2**

(A) All peaks for the 5 TFs were converted to the mouse genome (mm9). For the mapped peaks it was next determined whether these regions were also bound by the same transcription factor in mouse HPC-7 cells. Depicted are the percentage of the total number of peaks in our human megakaryocyte data that could be mapped to the mouse genome and what percentage of these are bound or not bound by the same TFs in HPC-7. Human megakaryocyte GATA1 and GATA2 peaks were compared with HPC-7 GATA2 data as no GATA1 data is available.

(B) The same analysis as for (A) was done for the 31 binding patterns of the 5 TFs.

| | |
|---|---|
| **Gata1** | 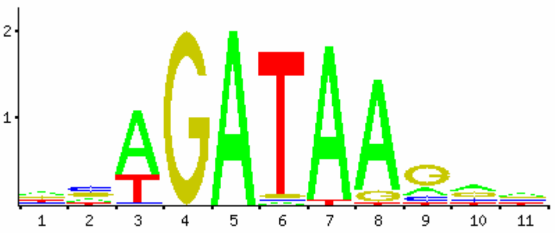 |
| **ETS1** | 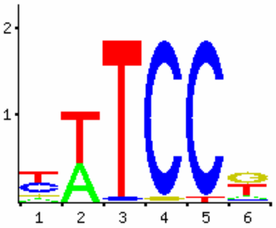 |
| **TAL1::TCF3** | 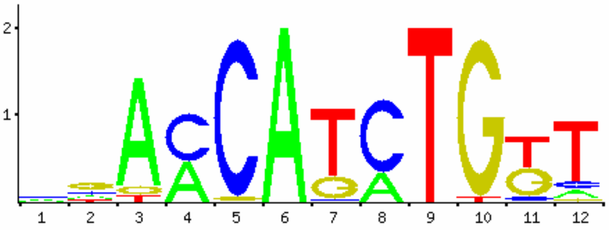 |
| **Runx1** | 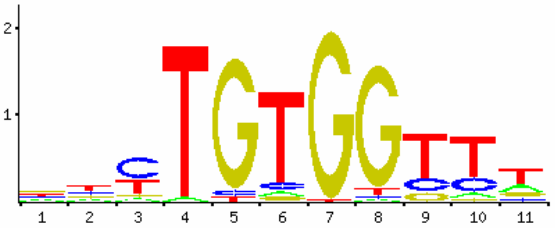 |
| **Tal1::Gata1** | 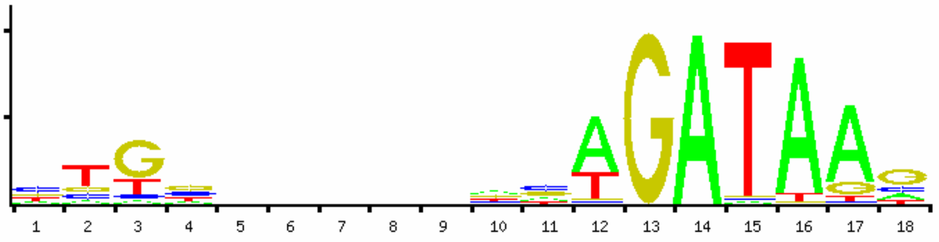 |

**Tijssen et al Supplemental Figure 2**

**Figure S2. Consensus Binding Sites from the JASPAR Database for the TFs Examined, related to Figure 3**

(A) GATA1

(B) RUNX1

(C) ETS1 (FLI1)

(D) E-box (SCL)

(E) SCL::GATA1

A

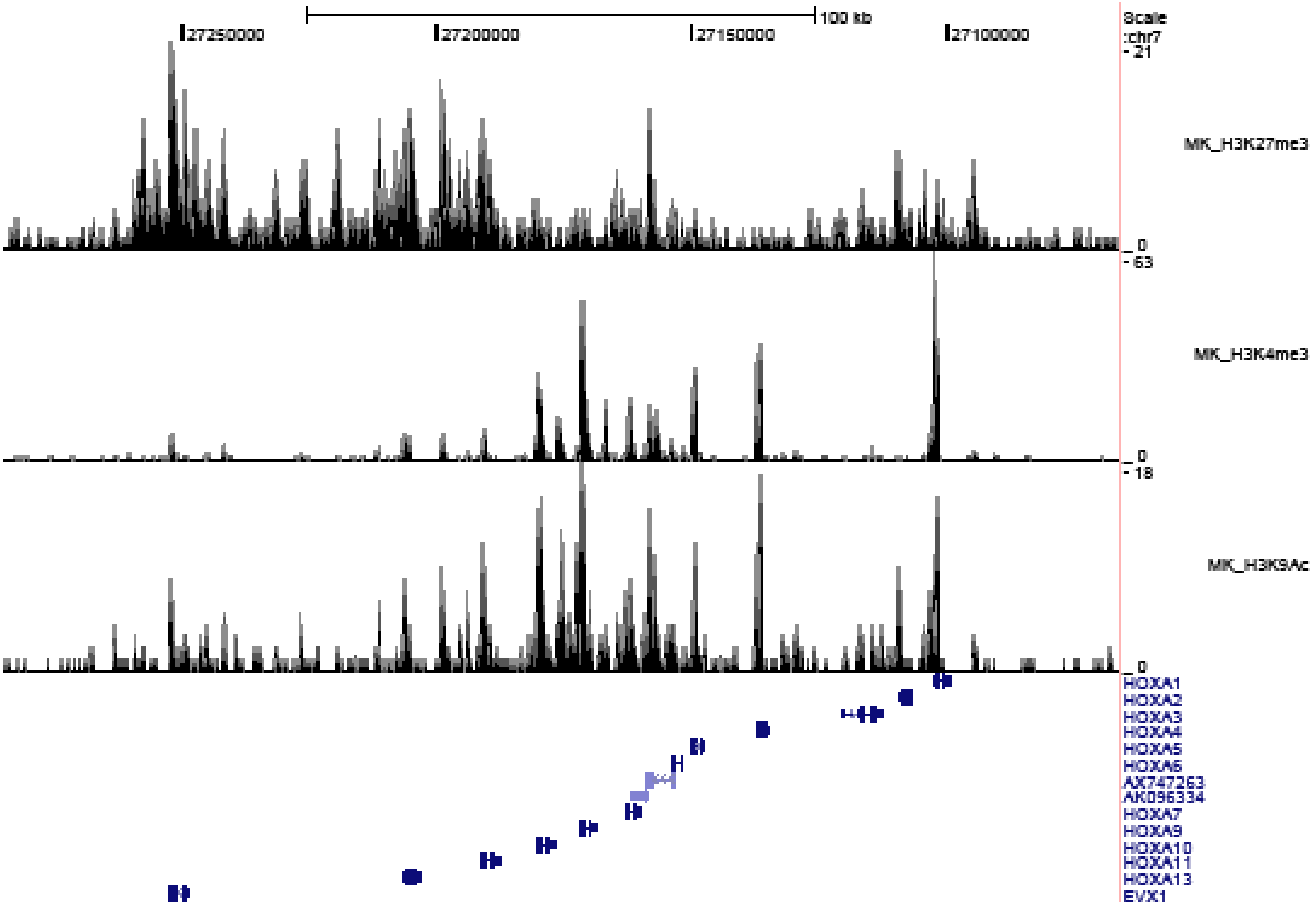


Tijssen et al Supplemental Figure 3

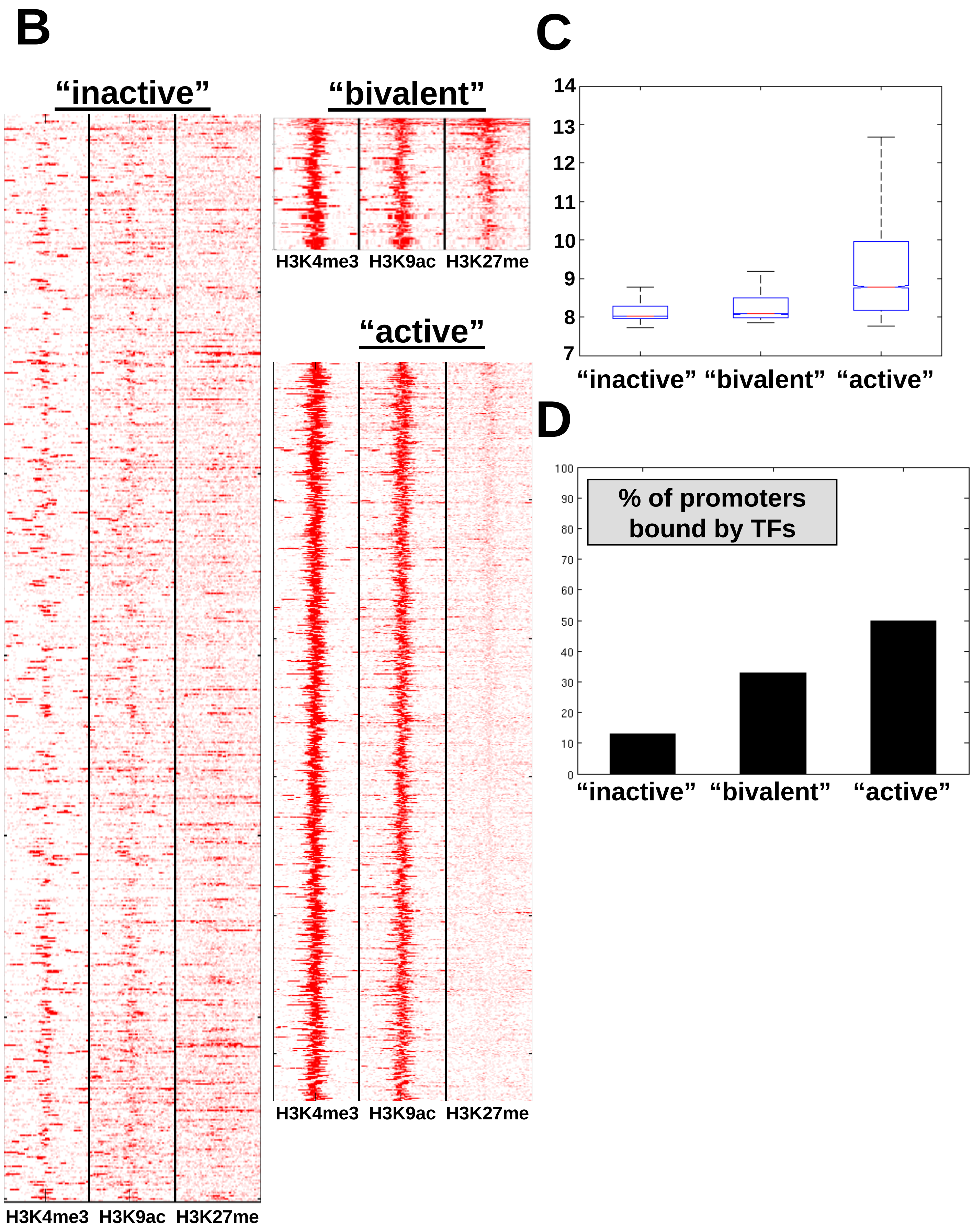


**Tijssen et al Supplemental Figure 3**

**Figure S3. Histone Modifications in the Human Megakaryocyte Genome, related to Figure 4**

Megakaryocytes were cultured as before (data not shown).

(A) Patterns for H3K27me3, H3K4me3 and H3K9ac around the HOXA loci show active genes on the right hand side with repressive marks on the left.

(B) A list of all human promoters was obtained from the mammalian promoter database. ChIP-Seq enrichment across 10 kb regions centered on all promoter sequences was determined, partitioned using Seqminer (Ye et al., 2010) and displayed as “heatmaps”, which allowed us to categorize promoters into “active”, “bivalent” and “inactive” in human megakaryocytes.

(C) Analysis of gene expression levels in megakaryocytes (Haematlas (Watkins et al., 2009)) shows the highest level of expression for genes with “active” promoters (log2 transformed gene expression scores are displayed as Boxplots).

(D) Binding of GATA1, GATA2, RUNX1, FLI1 and SCL is most prevalent for promoters “active” in MKs, with intermediate levels seen for “bivalent” promoters.

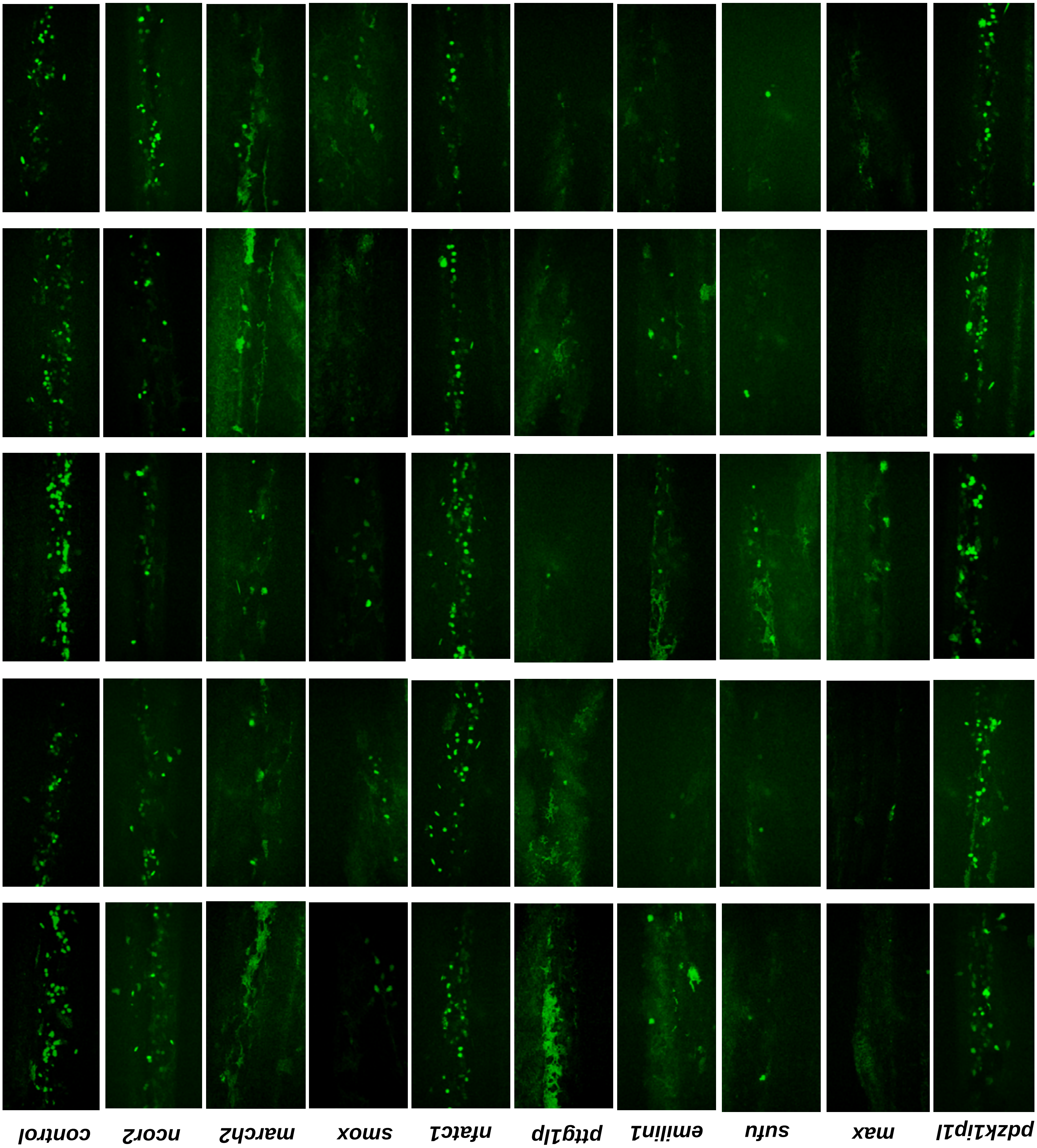


# Tijssen et al Supplemental Figure 4

**Figure S4. Morpholino Screen in *cd41* Transgenic Zebrafish Embryos, related to Figure 5**

Representative pictures at 72 hpf of 5 control transgenic *Tg(cd41:EGFP)* zebrafish embryos or injected with MOs. The GFP positive cells represent the number of presumed hematopoietic stem cells (*cd41low*) and thrombocytes (*cd41high*). For *march2* (n=44), *max* (n=63), *smox* (n=60), *pttg1lp* (n=50), *emilin1* (n=65) and *sufu* (n=65), a severe decrease of *cd41* positive cells was observed. *Ncor2* (n=53) depletion resulted in a mild phenotype, and *pdzk1ip1l* (n=49) and *nfatc1* (n=60) MO injected embryos showed no phenotype.

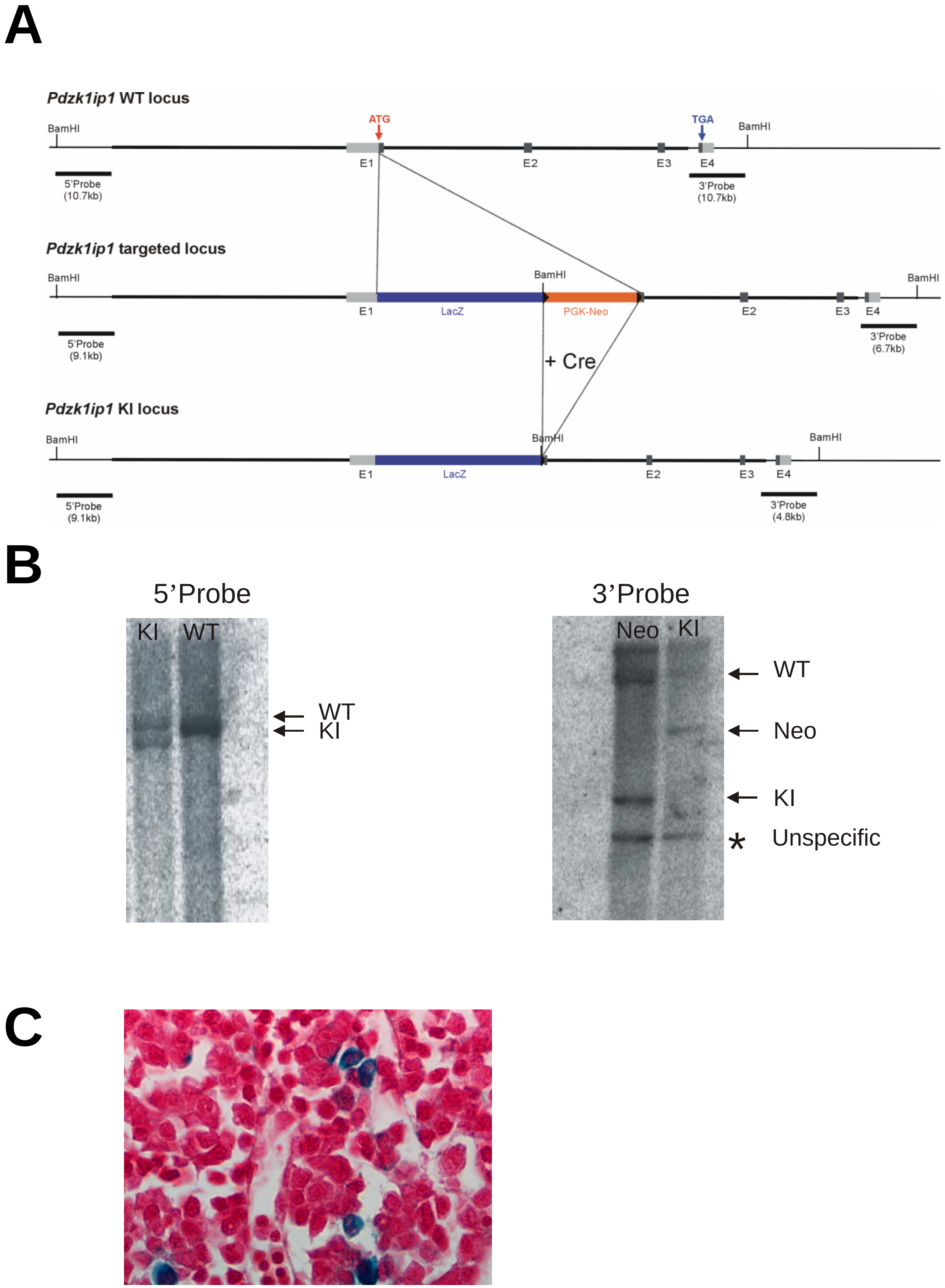


**Tijssen et al Supplemental Figure 5**

**Figure S5. Disruption of the *Pdzk1ip1* locus by homologous recombination, related to Figure 6**

(A) The structure of the wild-type allele of the murine *Pdzk1ip1* locus is illustrated with exons as grey boxes (untranslated regions in light grey and translated regions in dark grey). In the targeted allele, 131bp of *Pdzk1ip1* exon 1 (containing the ATG site) was deleted and a cassette containing the LacZ gene and a LoxP-PGK-Neo-LoxP cassete were introduced by recombineering. The Knock-in allele was generated by deletion of the LoxP-PGK-Neo-LoxP cassette using Cre recombinase. BamHI sites and probes used for Southern blot analysis are shown. The predicted band size for each probe is indicated.

(B) Representative Southern blots obtained with 5'probe (left panel) and 3' probe (right panel). WT- AB2.2 ES cells; Neo-*Pdzk1ip1* targeted ES cells; KI- Knock-in ES cells (Neo deleted); * unspecific band.

(C) Fetal liver cells of day 12.5 transgenic mouse embryos with the +19 enhancer fused to the *Pdzk1ip1* promoter. The lacZ positive cells in this picture have double lobed nuclei characteristic for megakaryocytes at this stage of embryogenesis (Matsumura and Sasaki, 1989).

| Transcriptional Control | Signal Transduction | Other | | No GO Annotation | |
|---|---|---|---|---|---|
| CBFA2T3 | BMP2K | ABCC1 | OAT | ANKRD33B | HTT |
| CDK5RAP2 | CD84 | ACSF3 | PDE3A | ASB8 | KIAA0125 |
| CDK9 | CDKL1 | AP1M1 | PLA2G6 | BCL8 | LAPTM5 |
| ENO1 | CHN2 | ASS1 | RAB7A | C11orf24 | LOC100272217 |
| G3BP2 | CXCL2 | BCL2L1 | RCN1 | C12orf68 | LOC284805 |
| HFM1 | DIAPH1 | C6orf103 | RDH16 | C14orf70 | MDM1 |
| ILK | DUSP28 | CACNA1C | RHBDL3 | C1orf150 | MIR557 |
| KLF2 | FERMT3 | CCND3 | RNPEPL1 | C1orf212 | MIR663 |
| LYL1 | GNA12 | CDH23 | RPN1 | C1orf55 | MIR760 |
| MAX | GNB5 | COPZ1 | RSPO2 | C21orf121 | NCRNA00164 |
| MEX3B | GP1BB | CTNND2 | SEPT5 | C5orf15 | PDZK1IP1 |
| NCOR2 | GPR182 | DAB2IP | SEPT9 | C5orf56 | PRR5 |
| NEUROG1 | GRIK4 | DLK1 | SLC39A3 | C8orf75 | PTTG1IP |
| NFATC1 | IRS2 | DNAJB2 | SLC39A4 | CCDC19 | RSPH1 |
| NFE2 | ITGA2B | EFHC2 | SLCO5A1 | CEP68 | SAMD14 |
| PMF1 | LRP5 | EMILIN1 | SMOX | CUX1 | SLC43A3 |
| PPBPL2 | MACC1 | EPB49 | ST8SIA6 | CXADRP2 | TIFAB |
| PPIE | P2RX1 | EVL | STOM | DLG2 | TMEM129 |
| RERE | PLXNA4 | FNTB | SYNRG | FAM73B | TMEM64 |
| RUNX1 | PTGIR | GCNT1 | TAGLN2 | GJA4 | TMEM88 |
| RXRA | RAMP1 | GGTA1 | TNFAIP8 | GNG8 | TSPAN33 |
| SLBP | RGS9 | IMPDH2 | TPM4 | HAUS2 | UBASH3A |
| SUFU | SEMA4D | LIMS1 | UBE2H | HCCA2 | |
| TAL1 | SH2D3C | MARCH2 | UCP3 | | |
| | TNIK | MICAL3 | UMODL1 | | |
| | TREML2 | MYL4 | ZC3HC1 | | |
| | USP20 | MYRIP | ZMYND8 | | |
| | XCL2 | | | | |

# Tijssen et al Supplemental Table 4

**Table S4. Candidate Target Genes of GATA1, GATA2, RUNX1, FLI1 and SCL in Primary Human Megakaryocytes, related to Figure 5**

151 candidate target genes of the 144 regions bound by all 5 TFs separated in GO-annotation groups.

| Gene | Ensembl ID | Morpholino sequence 5'-3' | Primer Name | Sequence | Tm (C) | Product Size (bp) | gel |
|---|---|---|---|---|---|---|---|
| NCOR2 | ENSDART00000001092 | GTTATTCTGCGAGCACAGAAAATCA | NCOR2F1-2000 | AACTCTGAGAAGCAGATGCGT | 59.25 | 2000 | 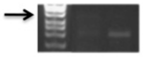 |
| | | | NCOR2R1-2000 | GGGGGTTCTGGAATATGGA | 59.55 | | |
| MARCH2 | ENSDART00000088419 | GAGCCACTGTTGAAAAATGACAAGT | MARCH2F162-1595 | TCTTTGTGGCTTGAGGTGTG | 59.87 | 1434 | 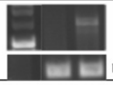 b-actin |
| | | | MARCH2R162-1595 | TGGCCCTGTAATACCCATGT | 60.07 | | |
| SMOX | ENSDARG00000036967 | TTTTACCTGTGGAGATAGGAAGGAA | SMOXF19-1981 | CGGTTTACGCTGAAGACGAT | 60.27 | 1970 | 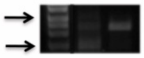 |
| | | | SMOXR19-1981 | CAGAAGGCACTTACAAAAATGG | 58.81 | | |
| NFATC1 | ENSDART00000052521 | CGCATCTGTAAGGTACAATCACATT | NFATC1F105-2238 | CGTTGTCATTTCTTGCGTTTT | 60.16 | 2134 | 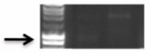 |
| | | | NFATC1R105-2238 | TTTGTGGGCAGGTATGTGAA | 59.96 | | |
| PTTG1IP | ENSDART00000058565 | GCAGTCTGTGAAAAGGACACATTTA | PTTG1IPF9-1063 | CCCAACTTCCAGCAGAAAAC | 59.71 | 1037 | 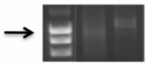 |
| | | | PTTG1IPR9-1063 | GGTGCACAAAACAAATCACAA | 59.48 | | |
| EMILIN1 | ENSDART00000035612 | AGTCCGATACCTGTGGTGAGATATT | EMILIN1F323-1346 | CCACTCTATTGGCGCTGATT | 60.24 | 1024 | 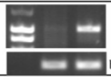 b-actin |
| | | | EMILIN1R323-1346 | CGACCTCCATTGCCATTACT | 59.96 | | |
| PDZK1IP1I | ENSDART00000012604 | CCGCAGTCTCTGAAAGAAACACAAA | PDZK1IP1IF50-1111 | TGGGAAAACCAGTCTCTGTG | 58.69 | 1062 | 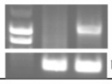 b-actin |
| | | | PDZK1IP1IR50-1111 | CCATTGAGACAATCCCCTTT | 58.84 | | |

| Gene | Ensembl ID | Morpholino sequence 5'-3' |
|---|---|---|
| MAX | ENSDART00000044462 | ATATCATCGTTGTCGCTCATTCTTC |
| SUFU | ENSDART00000079356 | CGCTGCTAGGCCGCATCTCATCCAT |

# Tijssen et al Supplemental Table 5

**Table S5. Morpholino Sequences for Targeted Genes and Primers Used to Verify Their Efficiency, related to Figure 5**

mRNA levels of *ncor2, march2, smox*, *nfatc1*, *pttg1lp*, *emilin1* and *pdzk1ip1l* in MO-injected embryos (next to ladder) and control were measured by RT-PCR, from equal amounts of total RNA. Splice modifications are seen as a band shift, as indicated with the black arrows. Amplification of β-actin, was used as a control where MO injection resulted in mRNA degradation.

| Gene ID | Gene name | Function | Homology | MO knock-down Thrombocytes | MO knock-down Erythrocytes |
|---|---|---|---|---|---|
| ***NCOR2*** | nuclear receptor co-repressor 2 | Promotes chromatin condensation to mediate the transcriptional repression activity of certain nuclear receptors | 45% | ✓ | ✓ |
| ***MARCH2*** | membrane-associated ring finger (C3HC4) 2 | E3 ubiquitin-protein ligase involved in regulating endosomal trafficking | 77% | ✓ | ✓ |
| ***SMOX*** | spermine oxidase | Polyamine oxidase involved in the regulation of the intracellular polyamine concentration | 70% | ✓ | ✓ |
| ***NFATC1*** | nuclear factor of activated T-cells | Plays a role in the inducible expression of cytokine genes in T-cells. Also controls gene expression in embryonic cardiac cells. | 52% | ✗ | ✗ |
| ***PTTG1IP*** | pituitary tumor-transforming 1 interacting protein | Binds pituitary tumor-transforming gene 1 protein (PTTG1) facilitating PTTG1's nuclear translocation and potentiating PTTG1's transcriptional activation of fibroblast growth | 45% | ✓ | ✓ |
| ***EMILIN1*** | elastin microfibril interfacer 1 | Extracellular matrix glycoprotein that associates with elastin fibres at the interface between elastin and microfibrils | 33% | ✓ | ✓ |
| ***SUFU*** | suppressor of fused homolog | Negative regulator in the hedgehog signaling pathway | 80% | ✓ | ✓ |
| ***MAX*** | MYC associated factor X | Transcriptional regulator that forms homodimers and heterodimers. The MYC-MAX complex is a transcriptional activator while the MAD-MAX complex is a transcriptional repressor | 82% | ✓ | ✓ |
| ***PDZK1IP1*** | PDZ-domain containing protein 1 interacting protein 1 | May play an important role in tumor biology | 37% | ✗ | ✓ |

# Tijssen et al Supplemental Table 6

**Table S6. Candidate Target Genes of GATA1, GATA2, RUNX1, FLI1, and SCL Selected for Knockdown in Zebrafish, related to Figure 5**

A short description of the function of the genes and the percentage of homology between human and zebrafish is given. A summary of phenotypes of MO knock down in zebrafish is depicted in the last two columns.

# Tijssen et al Supplemental Table 7

| | control | *ncor2* | *march2* | *smox* | *nfatc1* | *pttg1ip* | *emilin1* | *sufu* | *max* | *pdzk1ip1l* |
|---|---|---|---|---|---|---|---|---|---|---|
| | 63 | 38 | 1 | 9 | 65 | 1 | 3 | 3 | 1 | 53 |
| | 49 | 37 | 3 | 10 | 50 | 3 | 3 | 4 | 1 | 41 |
| | 83 | 24 | 8 | 11 | 74 | 2 | 5 | 7 | 0 | 71 |
| | 70 | 26 | 3 | 5 | 48 | 6 | 6 | 4 | 0 | 58 |
| | 59 | 45 | 3 | 11 | 50 | 4 | 10 | 2 | 2 | 63 |
| | 67 | 24 | 0 | 12 | 47 | 7 | 3 | 2 | 0 | 67 |
| | 51 | 32 | 3 | 5 | 38 | 1 | 7 | 3 | 2 | 61 |
| | 77 | 31 | 8 | 8 | 68 | 5 | 6 | 4 | 1 | 48 |
| | 69 | 48 | 4 | 9 | 50 | 5 | 8 | 3 | 0 | 37 |
| | 67 | 32 | 4 | 10 | 57 | 4 | 6 | 5 | 1 | 78 |
| | 88 | 25 | 1 | 5 | 68 | 3 | 8 | 5 | 0 | 50 |
| | 68 | 48 | 4 | 11 | 64 | 0 | 5 | 2 | 1 | 58 |
| | 53 | 45 | 1 | 6 | 61 | 4 | 5 | 4 | 0 | 64 |
| | 63 | 32 | 5 | 9 | 72 | 0 | 8 | 0 | 0 | 58 |
| | 64 | 20 | 2 | 11 | 68 | 1 | 0 | 6 | 1 | 40 |
| | 50 | 30 | 2 | 10 | 60 | 3 | 4 | 3 | 4 | 51 |
| | 86 | 33 | 9 | 12 | 49 | 1 | 2 | 2 | 0 | 56 |
| | 54 | 36 | 0 | 12 | 50 | 4 | 4 | 4 | 1 | 74 |
| | 59 | 31 | 7 | 8 | 70 | 4 | 7 | 1 | 1 | 40 |
| | 56 | 43 | 4 | 10 | 39 | 6 | 8 | 4 | 0 | 76 |
| average/embryo | **64.8** | **34** | **3.6** | **9.2** | **57.4** | **3.2** | **5.4** | **3.4** | **0.8** | **57.2** |

**Table S7. Number of *cd41*-Positive Cells in 20 Control Embryos and 20 Embryos Treated with Morpholinos for Each of the Candidate Target Genes, related to Figure 5**
